# The Underlying Neurobiological Mechanisms of Psychosis: Focus on Neurotransmission Dysregulation, Neuroinflammation, Oxidative Stress, and Mitochondrial Dysfunction

**DOI:** 10.3390/antiox13060709

**Published:** 2024-06-12

**Authors:** Neha S. Rawani, Allen W. Chan, Serdar M. Dursun, Glen B. Baker

**Affiliations:** Neurochemical Research Unit and Bebensee Schizophrenia Research Unit, Department of Psychiatry and Neuroscience and Mental Health Institute, University of Alberta, Edmonton, AB T6G 2G3, Canada; nrawani@ualberta.ca (N.S.R.); awchan@ualberta.ca (A.W.C.); dursun@ualberta.ca (S.M.D.)

**Keywords:** glia, HPA axis, gut microbiome, oxidative stress, mitochondrial dysfunction

## Abstract

Psychosis, defined as a set of symptoms that results in a distorted sense of reality, is observed in several psychiatric disorders in addition to schizophrenia. This paper reviews the literature relevant to the underlying neurobiology of psychosis. The dopamine hypothesis has been a major influence in the study of the neurochemistry of psychosis and in development of antipsychotic drugs. However, it became clear early on that other factors must be involved in the dysfunction involved in psychosis. In the current review, it is reported how several of these factors, namely dysregulation of neurotransmitters [dopamine, serotonin, glutamate, and γ-aminobutyric acid (GABA)], neuroinflammation, glia (microglia, astrocytes, and oligodendrocytes), the hypothalamic–pituitary–adrenal axis, the gut microbiome, oxidative stress, and mitochondrial dysfunction contribute to psychosis and interact with one another. Research on psychosis has increased knowledge of the complexity of psychotic disorders. Potential new pharmacotherapies, including combinations of drugs (with pre- and probiotics in some cases) affecting several of the factors mentioned above, have been suggested. Similarly, several putative biomarkers, particularly those related to the immune system, have been proposed. Future research on both pharmacotherapy and biomarkers will require better-designed studies conducted on an all stages of psychotic disorders and must consider confounders such as sex differences and comorbidity.

## 1. Introduction

Psychosis is an amalgamation of clinical symptoms that generically describes a mental state involving the loss of contact with reality [1]. Although the term itself lacks a unified definition and may vary across the literature, it generally describes a clinical construct of specific symptoms, namely. delusions, hallucinations, and disordered thinking [2]. Psychosis is an inherent characteristic of psychiatric disorders under the categorization ‘schizophrenia spectrum and other psychotic disorders’ seen in the Diagnostic and Statistical Manual of Mental Disorders, 5th edition—Text Revision (DSM-5-TR) [1,3]. It has been reported that 1.5–3.5% of the general population will meet the diagnostic criteria for a psychotic disorder, and it is estimated that around 3 in 100 individuals will experience at least one psychotic episode in their lifetime [2]. It is important to note that psychosis is not a nosological entity, but a set of clinical symptoms. The specifier “with psychosis” can be applied to depressive disorders as well [3]. 

Understanding the behavioral manifestations of psychosis has been difficult, given the heterogeneous nature of psychotic disorders. The symptoms are subjective and based on the patient’s individual perceptions. There is a need to more clearly establish the underlying neurobiology so that psychosis can be characterized through neuropathology as opposed to symptomology, which may differ from individual to individual. 

The most prevalent theory pertaining to the neurobiology of psychosis is that of a chemical imbalance in the brain, marked by dysregulation of the mesolimbic pathway as a result of dopaminergic hyperactivity [4]. As dopamine dysregulation is thought to be associated with several other psychiatric disorders without the onset of psychosis, there is a growing emphasis on exploring other mechanisms of physiological function instead of solely focusing on neurotransmitter interactions. Our current understanding of psychosis does not account for many of the emerging mechanisms or describe multiple interactions. Viewing psychosis through a neuroendocrine–immunomodulation lens, a perspective that addresses the interactive nature of communication between the nervous, endocrine, and immune systems, has indicated a multifactorial etiology and addresses some gaps in our understanding. A key limitation of this topic is that it is difficult to conclusively determine causation, given the interactive influences exerted between the different physiological mechanisms of interest. 

**Figure 1:**
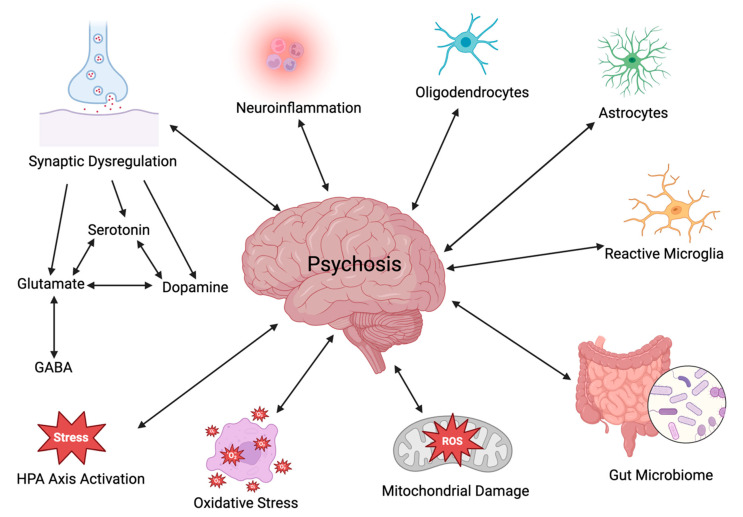
Potential interacting factors in the etiology of psychosis (created with BioRender.com).

Exploring the underlying neurobiological mechanisms that contribute to psychosis is of utmost interest and importance. Particular attention will be paid in the current review paper to several areas of interest, including neurotransmitter dysregulation, neuroinflammation, the hypothalamic–pituitary–adrenal (HPA) axis, the gut–brain axis (GBA), oxidative stress, and mitochondrial dysfunction (Figure 1). To obtain information on the neurobiological mechanisms underlying psychosis, a literature review was performed using PubMed, ScienceDirect, and MEDLINE for a period ranging from 2005 to 2024; relevant publications were obtained using the key search phrases dopamine, glutamate, serotonin, neuroinflammation, HPA axis, glial cells, gut-brain axis, oxidative stress, and mitochondrial dysfunction as they relate to psychosis. 

## 2. Neurotransmitter Dysregulation

### 2.1. Dopamine 

Although many hypotheses have tried to explain the neurobiological mechanisms underlying the onset of psychosis, the dopamine hypothesis remains a widely cited explanation. This hypothesis, based originally to a large extent on early pharmacological findings with antipsychotics [5,6,7], postulates that psychotic symptoms are attributed to the hyperactivity of dopaminergic neurons in the mesolimbic pathway [4]. The mesolimbic pathway is a dopaminergic pathway that starts from the ventral tegmental area (VTA) and projects to the nucleus accumbens [8]. The pathway also sends dopamine signals to other localized regions, including the amygdala, hippocampus, ventral and associative striatum, thalamus, and prefrontal cortex (PFC) [9]. Dopamine dysregulation within the associative striatum is behaviorally manifested as misappropriately attributing salience to certain stimuli which are clinically exhibited during delusions [9]. The underlying action of dopamine in the mesolimbic pathway in psychosis has been proposed to be hyperactivity seen at D2 dopamine receptors [8]. The original dopamine hypothesis has since been extended to take into account other symptoms of schizophrenia, such as negative symptoms and cognition, that do not seem to be explained by dopamine hyperactivity in the mesolimbic system [10,11,12]. 

There is pharmacological evidence, in addition to that of antipsychotics, that supports the involvement of dopamine in the pathophysiology of psychosis, since certain drugs with effects on dopamine are known to induce psychotic symptoms. In mouse models, psychostimulants that raise dopamine levels in the synaptic cleft produce behaviors associated with psychosis, and increased concentrations of D2 receptors have been observed in the striatum [13,14]. In human studies, psychostimulants such as amphetamines, cocaine, and cannabis induce postsynaptic sensitivity in the associative striatum, and abuse can cause auditory hallucinations and delusions [4,15]. It has been demonstrated that administering drugs such as amphetamines, which increase the functional availability of dopamine, can induce psychosis in healthy patients and worsen psychotic symptoms in schizophrenic patients [8,9,16]. 

One mechanism of action of cannabis is the stimulation of dopaminergic signaling in the nucleus accumbens of the mesolimbic circuitry, and the frequency and dose of cannabis use have been reported to correlate with the risk of developing psychosis [17]. Murray et al. [15] reported that 10 of 13 longitudinal studies they reviewed demonstrated that cannabis use is positively correlated with increased risk of subsequent development of psychosis [15]. These researchers concluded that heavy cannabis use leads to increased risk of psychosis, particularly when use starts in the early teens [15]. The pharmacological ability to induce acute psychosis has been reflected nosologically as a separate disorder, and the criteria for substance/medication-induced psychotic disorder are listed in the DSM-5-TR, which also contains a table of the most often recognized drugs involved [3]. 

The dopamine hypothesis has had an important relevance for pharmacotherapy, since many of the antipsychotics developed and currently in use antagonize dopamine D2 receptors [18]. However, many of those antipsychotics are not effective in treating negative symptoms and cognitive deficits in schizophrenia [13]. Also, in some psychotic patients who are treatment-resistant, there is no apparent dopamine dysregulation [9]. 

Although the mesolimbic circuitry is directly implicated in goal-directed behavior, findings have also demonstrated that mesolimbic dopamine signalling also integrates homeostatic processes, responding to changes in the physiological state through regulation of the HPA axis [10]. This suggests an involvement of other factors in psychosis in addition to mesolimbic dopamine. It has been reported that while patients are experiencing hallucinations, there is increased activation of the thalamus, hippocampus, and striatum. Overactivation of the PFC and reduced deactivation of thalamic and striatal networks are seen in schizophrenic patients experiencing delusions [9]. Additionally, focal brain lesions can induce psychotic symptoms, such as hallucinations, without apparently impairing normal subcortical dopamine function in areas of the mesolimbic circuitry [9]. 

Kesby et al. [9] have cautioned about the translation of dopamine research from animal models to clinical studies [9]. They have noted that studies on schizophrenia patients and on subjects with a high risk for developing schizophrenia have revealed increased presynaptic dopamine function in the associative striatum rather than, as would be assumed from animal studies, the limbic striatum [9]. They have also commented on shortcomings in some behavioral tests currently utilized in animal studies as representative of psychotic phenotypes in humans and have stated that the evidence to date suggests a network dysfunction in psychosis that includes several brain regions and multiple neurotransmitters [9]. In a later paper, Kesby and Turner [19] describe how more sophisticated techniques such as circuit-based optogenetics are very useful for studying neural processes underlying cognitive deficits in preclinical models. They give as an example a recent study by Tranter et al. [20] in which optogenetics was used in a postnatal phencyclidine (PCP)-treated rat model that produces schizophrenia-relevant cognitive impairments.

### 2.2. Glutamate 

Although the dopamine hypothesis remains an important theory of psychosis which has also been valuable in the development of antipsychotic drugs, it became clear that other factors are also important in the etiology of psychosis, including dysregulation of other neurotransmitters in addition to dopamine [4]. The glutamate hypothesis proposes that symptoms of schizophrenia and cognitive impairments are the result of the hypofunction of N-methyl-D-aspartate receptors (NMDARs) on γ-aminobutyric acid (GABA) interneurons in the cerebral cortex, resulting in overactivation of downstream glutamate signaling to the VTA and, possibly, consequent dopamine release in the mesolimbic system [4,21,22].

Buck et al. [13] proposed that a subpopulation of neurons that could co-release dopamine and glutamate may be implicated in the abnormal reward-seeking behaviors seen in schizophrenia and in individuals at high risk for psychosis. 

Although there is evidence to suggest that the dopamine–glutamate interplay is key to disrupted synaptic communication, there is some dispute regarding directionality. Increased synaptic cleft concentrations of dopamine, such as those produced acutely by amphetamines, may result in increased glutamate signaling, suggesting glutamate dysregulation is a downstream consequence [13,23] which may promote excitotoxicity and consequent neuronal damage [23]. However, it has also been shown that dopamine hyperactivity can be a downstream consequence of high levels of glutamate release [4]. 

Aryutova et al. [18] have provided a table of findings by several research groups on the etiology of psychosis and have listed several pharmacological studies supporting interactions between dopaminergic and glutamatergic systems. Mice with m-Glu2/3 glutamate receptors knocked out exhibit super-sensitivity to dopamine, providing further evidence of crosstalk between dopamine and glutamate [24]. NMDAR antagonists such as ketamine are known clinically to induce symptoms characteristic of psychosis [13,25,26]. The bidirectional crosstalk between glutamate and dopamine is demonstrated by a study in which the antipsychotic haloperidol, a potent D2 receptor antagonist, counteracted the processing negativity of the NMDAR antagonist ketamine [27]. Treating schizophrenic patients using the antipsychotic drug clozapine also blunts ketamine-induced psychosis [25,28].

A possible downstream consequence of the elevated release of glutamate in psychosis is excitotoxicity. Normal functional communication in the brain relies on a balance between excitatory and inhibitory networks, utilizing glutamate and GABA as neurotransmitters, respectively, and a perturbance of the excitatory/inhibitory (E/I) balance contributes to neural network dysfunction and leads to cognitive and behavioral deficits. Abnormalities in GABA neurotransmission are also linked to the effects of hypoactive NMDARs on GABA interneurons, leading to E/I imbalance as a possible mechanism of increased dopamine activity and, ultimately, psychosis [29]. 

Regarding the involvement of glutamate in psychosis, there has also been a great deal of interest in recent years in the possible role of the amino acid D-serine, a potent co-agonist at the glycine site on the NMDAR. Abnormalities in the levels of D-serine and/or the activity of enzymes involved in its synthesis and catabolism have been proposed in schizophrenia [30], and D-serine and inhibition of its catabolism have been reported to be useful in treatment of schizophrenia [22,31,32]

### 2.3. Serotonin 

There is evidence that the neurotransmitter serotonin (5-hydroxytryptamine; 5-HT) is implicated in psychosis pathophysiology. The serotonin hypothesis implicates excessive activation of 5-HT2A receptors [33], particularly those on glutamate neurons in the anterior cingulate cortex and dorsolateral frontal lobe [34] in psychosis. Upregulation of these 5-HT2A receptors may lead to excess glutamate release in the cortex [4]. Therapeutic interventions have demonstrated that the antagonism of serotonin 5-HT2A receptors is effective for reducing psychotic symptoms in Parkinson’s disease [4].

Several atypical (second-generation) antipsychotics block both dopamine D2 and 5-HT2A receptors, but they vary markedly in their effectiveness in blocking the psychotic effects of methamphetamine [35]. It has been proposed that hallucinogenic drugs such as psilocybin and lysergic acid diethylamide (LSD) act as 5-HT2A receptor agonists [4]. Gonzalez-Maeso et al. [36] proposed that these hallucinogenic drugs can interact with both glutamate and serotonin receptors at what has been proposed as the 5-HT2AR/mGluR2 complex. Analysis of post-mortem brain tissue of untreated schizophrenia patients has revealed upregulation of 5-HT2A receptors and downregulation of mGluR2 glutamate receptors [36]. It has been hypothesized that this complex integrates serotonin and glutamate signaling to regulate sensory gating (the ability of the brain to modulate sensitivity to sensory stimuli that are incoming), a perturbation of which has been proposed to occur in psychosis [36]. 

Psychosis may also involve interactions with other physiological mechanisms such as neuroinflammation, oxidative stress, and mitochondrial dysfunction, which are linked to the inhibition of neurogenesis and the onset of cell death, so exploring these facets in the context of psychosis has also been an active area of research. 

## 3. Neuroinflammation

As the brain is considered immunologically privileged due to the presence of the blood–brain barrier (BBB), the consequences of neuro-immune interactions have often been overlooked. However, considerable preclinical and clinical evidence now points to significant neural–immune interactions and a bidirectional communication between the nervous and immune systems. Arguably, inflammation is a double-edged sword. While an inflammatory response is a protective mechanism, unaddressed chronic inflammation can be harmful, making it an underlying mechanism of action for neuropathology. An inflammatory response characterized by elevated pro-inflammatory cytokine concentrations is characteristic of many psychiatric disorders, and the finding that neuroinflammation tends to precede psychosis symptomology implicates it as part of the psychotic pathogenesis [37]. 

Pro-inflammatory cytokines such as interleukin (IL)-6, IL-8, and tumor necrosis factor (TNF)-α are involved in the perpetuation and maintenance of an inflammatory state. Low levels of cytokine production are integral to maintaining homeostasis, but the severity of clinical symptoms in schizophrenia has been reported to correlate with the levels of pro-inflammatory cytokines [38]. Clinical studies have reported increased concentrations of IL-β, IL-6, and TGF-β in chronic schizophrenia and first-episode psychosis patients [39]. Similarly, analysis of post-mortem brain tissue revealed that 40% of schizophrenia patients had a high inflammatory-expression signature [40]. It has also been reported that increased concentrations of pro-inflammatory cytokines were seen not only in first episode psychosis patients, but in young at-risk adults prior to the onset of psychosis [41,42], and it has been hypothesized that these cytokines may be useful trait markers for psychosis [41]. A recent clinical study by Kim et al. [43] also examined the longitudinal relationship between peripheral pro-inflammatory cytokines and symptomology in first-episode schizophrenia patients, and the results revealed a strong correlation between cytokine concentration and symptomology [43]. The chronic use of methamphetamine has also been linked to a perturbed immune homeostasis characterized by increased pro-inflammatory cytokine concentrations [44]. 

These findings collectively point toward the role of pro-inflammatory cytokines with respect to psychosis. Elevated concentrations of these cytokines are implicated in chronic immune activation which disturbs the physiological pathways involved in neurotransmission. Miller and Goldsmith [45], in a comprehensive review, summarized the evidence of immune dysfunction across the clinical course in schizophrenia. They also reviewed the mixed success of adjunctive anti-inflammatory agents and other immunomodulatory treatments in schizophrenia and suggested that adjunctive monoclonal antibody immunotherapy should be considered in the future. Clinical trials with a number of monoclonal antibodies in schizophrenia are ongoing; the initial results are potentially promising, but the need for longer-term treatment and larger cohorts in future investigations is emphasized [46,47].

Both dopamine and glutamate have been found to be involved in inflammation-mediated processes. As studies have demonstrated that induced IL-1β promotes conversion of rat mesencephalic progenitor cells into dopamine neurons, it has been argued that IL-1β has a causal role in dopamine dysregulation [48,49]. Other studies have demonstrated that the administration of IL-8 and IL-1β in infancy to rodents affects dopaminergic signaling in adulthood [49,50,51]. Other pro-inflammatory cytokines have also been linked to dopaminergic signaling. For example, Felger et al. [52] demonstrated that the repeated administration of the cytokine interferon (IFN-α) in rhesus monkeys results in altered dopamine release in the striatum [52]. Furthermore, as dopamine is involved in the dynamic regulation of immune cells, the cytokine–dopamine interactions are believed to be bidirectional [53]. In a comprehensive review paper, de Bartolomeis et al. [49] reported that a link between inflammation and glutamate was supported by studies on overactive microglia in schizophrenia patients and maternal immune activation (MIA) animal models. The immune response of glial cells can cause an impairment of glutamate reuptake and increased production of kynurenic acid (an antagonist of NMDA receptors which can reproduce the NMDAR hypofunction characteristic of psychosis). 

Glial cells are known to contribute significantly to inflammation and can also affect other mechanisms that may contribute to psychosis. Therefore, a discussion of some of those effects follows (see Table 1 for a summary).

### 3.1. Microglia 

Microglia reside in the central nervous system (CNS) and are immune cells that facilitate inflammation in their active state. In normal physiological conditions, microglia provide ongoing immune surveillance and are the first line of defense against harmful cellular debris. However, in pathological conditions, such as psychosis, microglia become chronically active, thus facilitating a state of inflammation [54].

Pro-inflammatory cytokines can trigger the conversion of microglia from a resting to an active phenotype in which these cells can also release pro-inflammatory cytokines such as IL-6 [49,55]. Microglia-derived cytokines also reciprocally influence and modulate neuronal function [55].

Increased microglial activation has been reported in schizophrenia patients [56]. Furthermore, elevated microglial activity and neuroinflammation have been reported to be correlated with psychosis, demonstrating a positive correlation with symptom severity [56]. Chronic microglial activation and elevated pro-inflammatory markers precede the onset of psychosis, demonstrating that an inflammatory status may be predictive of psychotic symptoms [57]. Chronic microglial activation has been identified as a mechanism behind excessive synaptic pruning, loss of brain volume in the cortex and cellular dysfunction in the PFC, all of which are characteristic pathologies of psychosis in schizophrenia [49]. Activated microglia, through secretion of reactive active species (ROS) and pro-inflammatory cytokines, can disrupt blood–brain barrier (BBB) endothelial function [54].

MIA models in rodents have shown that a maternal infection may lead to immunological changes that mobilize microglia, thus predisposing offspring to psychosis-like symptoms [49,57]. An animal inflammatory model using MIA demonstrated behavioral abnormalities consistent with schizophrenia as well as increased dopamine in the PFC and nucleus accumbens [49]. These findings demonstrate that psychosocial stressors can cause microglial activation, resulting in a low-grade inflammatory state. The effects of MIA on microglia are evident at a morphological level through changes in microglial density and arborization, potentially further contributing to the pathogenesis of psychosis [49]. Furthermore, recent findings suggest that antipsychotics reduce microglial activation, offering an additional reason as to why antipsychotics may be of assistance in the clinical setting [55]. 

Glutamate is also thought to interact with microglial cells as a driver of dendritic apoptosis and synaptic pruning in psychosis [58]. Pharmacological models of schizophrenia include the application of NMDAR antagonists, such as phencyclidine (PCP), ketamine, and dizocilpine (MK-801). Such models have resulted in neuroinflammation characterized by microglial reactivity and pro-inflammatory cytokine production and apoptosis [59]. Glutamate excitotoxicity is also characterized by an overproduction of pro-inflammatory cytokines and neuroinflammation through its action on microglia [60].

### 3.2. Astrocytes 

Astrocytes, the most abundant glial cells in the CNS, are multi-faceted cells that express a wide range of receptors, transporters, enzymes, and ion channels [61].

They are key to maintaining homeostasis in the CNS by regulating the following: provision of nutrition to neurons; neurotransmitters (particularly glutamate); ion and water homeostasis; formation and modulation of synapses; cerebral blood flow and metabolism; development, maintenance and function of the blood–brain barrier (BBB); iron transport; and defense against oxidative stress [61,62,63,64]. Astrocytes transfer glucose and lactate to neurons and can remove neurotransmitters such as glutamate from the synaptic cleft and release modulatory factors [64]. It has been reported that astrocytes are responsible for retrieving approximately 80% of the glutamate from the synaptic cleft via glutamate transporters [65]. Astrocytes also express GABA-A and GABA-B receptors as well as GAT-1 and GAT-3 transporters and can release GABA and regulate its concentration [66]. In addition to mediating the uptake and release of neurotransmitters, astrocytes can promote the function of synapses by the secretion of synaptogenic and neurotrophic factors such as thrombospondins, hevin, and transforming growth factor-beta1 (TGF-β1) [66]. Astrocytes can also eliminate synapses by various mechanisms, including direct phagocytosis, stimulating microglia to phagocytose and activating the intracellular inositol 1,4,5-triphosphate (IP_3_) pathway, resulting in release of Ca^2+^ from the endoplasmic reticulum [61]. Astrocytes are also an important component of the glutamate–glutamine cycle. In this cycle, glutamate released from neurons is transported into astrocytes, where it converted to glutamine, which is then returned to neurons, where it is subsequently converted to glutamate [67,68]. Analysis of CSF has demonstrated that first-episode psychosis (FEP) and drug-naïve schizophrenia patients have an elevated glutamine-to-glutamate ratio [69,70]. The density of astrocytes expressing the disrupted-in-schizophrenia 1 (DISC1) gene has been reported to be decreased in the dentate gyrus of hippocampus in schizophrenia patients compared to healthy controls [71]. This situation will result in decreased synthesis of the NMDAR co-agonist D-serine [22]. 

Although there is considerable disagreement in the literature, several postmortem studies on schizophrenia have reported changes in astrocytic density and/or astrocytic markers in schizophrenia [61]. Laricchiuta et al. [72] discuss the confounding factors that make clinical studies on astrocytic and microglial markers in schizophrenia difficult. 

Astrocytes can also play an important role in oxidative stress and neuroinflammation. As indicated by Chen et al. [63], the actions of astrocytes regarding oxidative stress can be a mixed blessing. Under normal circumstances, astrocytes can have an antioxidant response, producing various antioxidants (e.g., glutathione), removing glutamate, and activating antioxidant systems like Nrf2, protecting against the damage related to oxidative stress. However, under pathological conditions, astrocytes can be a source of reactive oxygen species (ROS) or reactive nitrogen species (RNS) as a result of mitochondrial dysfunction, impaired metabolism, increased glutamate, and/or reduced antioxidant generation. These free radicals promote activation of microglia and neuroinflammation [63]. Oxidative stress can also have a detrimental effect on astrocytes. It is known that oxidative stress can affect the metabolism and secretion of glutamate in astrocytes [63]. For example, hydrogen peroxide can cause mitochondrial damage [73] and reduce the effectiveness of the glutamate transporters of astrocytes [63]. 

As indicated above, astrocytes affect the development, maintenance and function of the BBB. It is now well documented that there is often BBB dysfunction in psychosis [54]. Najjar et al. [74] reported neurovascular epithelial dysregulation and BBB hyper-permeability in schizophrenia patients. The functional impairment of the BBB, described as ‘leaky’ in the literature, is of interest regarding both neuropathology and pharmacological interventions in psychosis. It has been postulated that BBB disruption is secondary to neuroinflammation, and the leaky BBB allows for a greater infiltration of pro-inflammatory mediators, worsening the BBB integrity and inflammatory state [54,75].

The endothelium of the BBB acts within the modular neurovascular unit comprising a capillary segment and its associated pericytes, basement membranes, astrocytes, microglia, and neurons [54,76]. Changes in the BBB neurovascular unit may include the following: alterations in the expression of ion channels and drug transporters on endothelial cells and glia; increased leakiness of tight junctions; and changes in regulation of adhesion molecules and leucocytes. See [54] for tables on abnormalities of BBB-associated molecules in psychotic disorders and the effects of risk factors for psychotic disorders on the BBB. The acidic calcium-binding protein S100B is secreted by astrocytes and oligodendrocytes. Although its validity as a marker of BBB disruption has been questioned [77], meta-analyses have reported that schizophrenia patients have higher serum S100B concentrations than controls [78,79] and that there is a positive correlation between S100B levels and positive symptoms, total psychopathology scores, and duration of psychotic illness [79]. As mentioned previously in this review, there is considerable evidence supporting immune dysfunction in psychotic disorders, and activated microglia, through secretion of ROS and cytokines, may disrupt BBB endothelial function [54]. Astrocytes can have a dual role in BBB function since several astrocyte-derived vascular permeability factors can aggravate BBB disruption. Several protective factors, also astrocyte-derived, can attenuate the increase in BBB permeability, resulting in BBB protection [80]. According to Pollak et al. [54], the increased glutamate release as a result of hypofunctioning NMDARs in psychosis may also contribute to a leaky BBB.

### 3.3. Oligodendrocytes

Psychosis symptoms have been identified in clinical conditions that display a disruption of normal myelination, pointing towards abnormal oligodendrocyte function. In normal physiological conditions, oligodendrocytes are the myelinating cells of the CNS, and thus are critical for the propagation of action potentials and neuronal communication. 

Post-mortem analysis of brain tissue of schizophrenia patients revealed that approximately 14–22% had reduced densities of oligodendrocytes [81]. Abnormal myelination of connecting fibers in the left front-temporal region, a brain region thought to be involved in the development of auditory and verbal hallucinations, was also seen in psychosis patients [82]. Furthermore, some studies have linked demyelination-induced delays in processing speed to discrepancies in sensory feedback mechanisms in patients with psychosis [81]. A study by Zhang et al. [83] found that PCP-injected mice not only displayed schizophrenia-like behaviors, but PCP administration led to impaired myelination in localized regions such as the frontal cortex and decreased quantities of oligodendrocytes. Myelin gene knockout mice also have schizophrenia-like behavioral deficits, leading to the postulation that abnormal oligodendrocyte function contributes to the etiology of psychotic disorders [81]. As patients with myelin-related disorders have also exhibited psychosis, this led to the postulation that perhaps interruptions of myelination in localized regions such as the frontotemporal, callosal and periventricular fiber tracts underlie psychotic behavior [84]. 

Oligodendrocytes are also susceptible to the excitotoxic effects of glutamate [84]. These cells are also thought to have immune–inflammatory functions, and thus can downregulate the extent of inflammatory damage [85,86]. This suggests that the loss of oligodendrocytes has a multifactorial effect, leading to impaired myelination, as well as permitting infiltration of pro-inflammatory cytokines and reactive microglia [86]. 

Crosstalk among microglia, astrocytes, and oligodendrocytes may play an important role with regard to psychosis. Although the oligodendrocytes are the glial cells primarily involved in myelination, it has been reported that microglia and astrocytes, in both their quiescent and activated forms, can modify differentiation of oligodendrocyte progenitor cells into myelinating oligodendrocytes and affect remyelination by oligodendrocytes [87]. Microglia and astrocytes interact with each other in multiple ways, including at the level of direct contact, and on secretion of factors affecting inflammation and exocytosis [88]. 

## 4. HPA Axis 

The HPA axis consists of a cascade of neuroendocrine interactions responsible for homeostatic regulation. Elevations in the activity of the axis have been implicated in several mental disorders, including schizophrenia and other psychotic disorders [89].

Stressors can lead to a perturbance of homeostasis through activation of the HPA cascade. Psychosocial stressors have been proposed as risk factors for subsequent development of psychotic illness [90]. Similarly, trauma history has been reported to be a predictor of psychosis, with the severity of adverse childhood experiences and trauma correlated with the risk of emerging psychotic symptoms [90]. Mayo et al. [90] hypothesized that repeated exposure to stressors leads to stress sensitization and, consequently, HPA axis dysregulation.

A state of low-grade inflammation, characterized by increased levels of pro-inflammatory cytokines such as IL-6, is correlated with childhood trauma and abuse [49]. Since psychological stress can also activate an innate immune response through HPA activation, elevation of levels of pro-inflammatory cytokines could be a consequence of chronic HPA activation. HPA axis dysregulation secondary to hypercortisolemia has been reported in psychotic patients, and the mechanism of action is believed to be increased concentrations of IL-1, IL-6, and TNF-α [49].

Glucocorticoids such as cortisol are key mediators of the HPA axis, and thus a potential biological marker of stress. In patients with psychosis, elevated cortisol levels have been observed where repeated exposure to stress results in both sensitization of the HPA axis and an increased risk of psychosis [90]. Stress-induced cortisol hypersecretion may contribute to psychosis symptomology. Kazi and Hoque [91] reported that administering corticosteroids to patients with no prior psychiatric history induced neuropsychiatric side effects such as acute psychosis. Additionally, increased release of glucocorticoids can also directly affect dopaminergic neurotransmission within the mesolimbic pathway. According to Mikulska et al. [92], studies support a causative link between elevated cortisol levels and increased dopamine release [92]. This is further supported by studies in which glucocorticoid antagonists relieve psychotic symptoms [92]. The finding that antipsychotics can suppress HPA axis activity suggests another mechanism through which antipsychotics are clinically effective [92]. 

HPA axis dysregulation, in the form of cortisol hypersecretion, has many downstream consequences that can exacerbate psychiatric symptoms. There is bidirectional communication between the HPA axis and systemic inflammation such that chronic disruption of homeostasis can contribute to psychotic symptoms [92]. Aligned with the stress-sensitization model, a lack of regulation implicates a heightened stress response, while the inability to respond to stressors increases ‘wear and tear’ in the body. Thus, unregulated HPA axis activity can be viewed as a pathway contributing to psychosis through neuroinflammation. The systematic consequences of stress-induced HPA dysregulation and elevated cortisol levels include alterations of microglial activity and, consequently, a state that resembles neuroinflammation [55].

The neural diathesis-stress model postulates that repeated exposure to psychosocial stressors leads to abnormalities in the HPA axis, which consequently exacerbates the response to stressors and leads to dysregulation of both dopamine and glutamate levels [93]. Homeostatic signals are also integrated by dopaminergic neurons of the mesolimbic circuitry [10]. Hormones related to physiological states such as hunger, fluid intake, and other satiety behaviors are expressed throughout the brain, and interact directly with the mesolimbic pathway [10]. Glutamate dysregulation drives HPA axis activity through increased excitatory signaling, whereas chronic HPA activity also alters glutamate homeostatic levels [93]. Thus, hormonal dysregulation may be relevant to the pathophysiology of psychotic disorders through interaction with neurotransmitters and HPA dysfunction [93]. 

## 5. Gut–Brain Axis 

**Figure 2:**
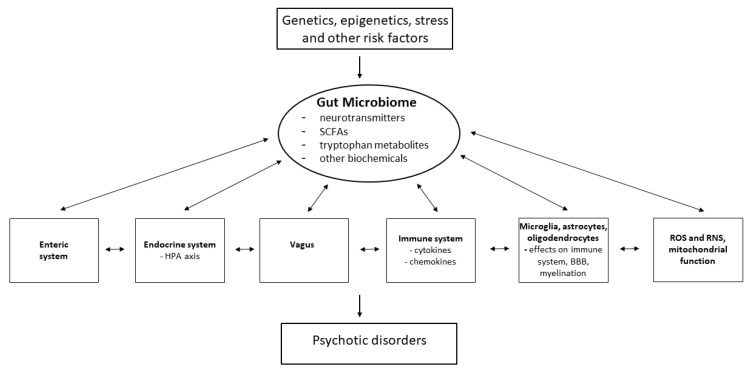
Potential interactions of the gut microbiome with other factors involved in psychosis. SCFAs = short chain fatty acids; ROS = reactive oxygen species; RNS = reactive nitrogen species. Adapted with permission from Refs. [98,99]. Copyright 2013 Elsevier and 2024 Eureka Science/Bentham Science respectively.

The brain is also in constant communication with the gastrointestinal (GI) system through the GBA, a bidirectional communication network, the effects of which are exerted through pathways including the neuroendocrine HPA axis, the immune system, and the autonomic nervous system (e.g., the vagus nerve) [94,95,96] (Figure 2). Psychosis is also often associated with chronic GI inflammation, suggesting that dysfunction in psychosis may extend to changes in the gut microbiome, which has critical functions involved in the regulation of host homeostasis. Myelination, neurotransmission, BBB organization, the HPA axis and neural–immune interactions can be directly altered as a consequence of the activity of the gut microbiome [95,96,97,98,99]. 

Kraeuter et al. [100], in a systematic review of preclinical and clinical studies in psychosis, concluded that despite variable findings in the literature due to complicating factors such as lifestyle/environment, sex, gender, age, details of methods for processing fecal samples, and medication effects, the research overall supports the hypothesis of an alteration in the gut microbiome in psychosis, compared to healthy controls, and that the gut microbiome changes may precede the appearance of clinical diagnostic symptoms of schizophrenia-spectrum disorders. Zhu et al. [101] found that when *Streptococcus vestibularis* bacterium, one of 11 different bacterial taxa reported by them to be enhanced in medication-free schizophrenia patients, as compared to controls, was administered to mice, altered neurotransmission and schizophrenia-like behavioral deficits were observed. Similarly, animals that received gut microbiota from schizophrenia patients through a fecal transplant had lower glutamate levels and higher glutamine and GABA levels in the hippocampus than did healthy controls and displayed a schizophrenia-like behavioral phenotype [102]. In a systematic review which included studies on gut microbiota composition in people with schizophrenia and healthy controls, McGuinness et al. [103] reported that 81% of the analyses found no difference in α-diversity (richness or evenness of the taxa) between the two groups, while 79% found a significant difference in β-diversity (variability in the identity of the taxa observed). 

The gut microbiota can produce inflammatory metabolites that cross the BBB, but inflammatory processes also influence the composition of the gut microbiome [104]. Inflammatory processes have been found to affect the permeability of the GI tract; abnormalities in the epithelial barrier are characteristic findings in psychotic disorders [97]. Individuals at clinical high risk (CHR) for psychosis have been reported to have increased plasma levels of pro-inflammatory cytokines, which was predictive of decreased volume in the PFC [97]. The same authors hypothesized that disturbed gut microbiome composition could be an etiological factor behind elevated levels of pro-inflammatory cytokines in the periphery and CSF [97]. 

The gut microbiome also plays a role in regulating the maturation and function of microglia [97,105]. A study by Erny et al. [106] found microglia defects in germ-free (GF) mice, which were partially restored through supplementation with short-chain fatty acids (SCFAs). SCFAs such as acetic acid, propionic acid, and butyric acid are by-products of certain gut microbes, can cross the BBB, and have been shown to decrease the reactivity of microglia, attenuating inflammation [107]. Gut microbiota dysregulation has also been linked to microglia-mediated neuroinflammation in schizophrenia [108]. A study by Zhao et al. [109] postulated that the gut microbiota modulates the maturation of astrocytes. Although these findings are not specific to psychosis, the interactive relationship between gut dysbiosis, BBB permeability, and astrocytic function is of great interest. Furthermore, the gut microbiome also influences myelination within the CNS. This was supported by evidence that GF mice exhibited abnormal myelination of neuronal axons within the PFC [96,110]. 

There is a bidirectional relationship between the HPA axis and the GBA. Not only can the gut microbiota influence HPA axis activity through mediators that cross the BBB, but exposure to stressors can affect mediators of the HPA axis and ultimately affect the GI barrier. Studies using GF mice have shown that exposure to gut microbes, particularly at an early developmental stage, is necessary for the development of the HPA stress response [94]. Studies have demonstrated that stress, in the form of MIA, results in chronic HPA axis activation and, consequently, changes in the composition of the gut microbiome in both rats and rhesus monkeys [111,112]. Stressors have been linked to altered quantities of *Lactobacillus*, and probiotic supplementation with *Lactobacillus helveticus* partially rectifies HPA dysfunction and suppresses hypersensitivity associated with visceral pain [112,113].

The gut microbiome can also catalyze synthesis of neurotransmitters such as dopamine, noradrenaline, glutamate, GABA, acetylcholine, and serotonin. The gut microbes produce large amounts of tryptophan (the precursor to serotonin) and related tryptophan metabolites such as kynurenic acid and quinolinic acid. These metabolites have been proposed as being involved in the etiology of several neuropsychiatric disorders and in oxidative stress and mitochondrial dysfunction [105,114]. Administration of antibiotics such as ampicillin has been linked to decreased dopamine levels in the hypothalamus [108,115]. Zheng et al. [102] transferred the microbiota of schizophrenia patients to mice, which induced changes in the gut microbial composition of the mice and produced a schizophrenia-like behavioral phenotype. Perturbed glutamatergic neurotransmission and notably decreased concentrations of glutamate in the hippocampus were observed as a result [102]. Furthermore, administering the prebiotic galacto-oligosaccharide (B-GOS) to rats increased NMDAR function in cortical regions [105]. With respect to serotonin, approximately 95% of its production is within the GI tract [105]. Intestinal microorganisms can affect the metabolism of tryptophan, a serotonin precursor obtained from dietary protein which can cross the BBB [107]. Plasma concentrations of tryptophan have been reported to be notably lowered in both first-episode psychosis and antipsychotic-free schizophrenia patients [116,117]. Lowered plasma tryptophan was found to be correlated with a lower fractional anisotropy of white matter and lower glutamate levels in the frontal white matter of schizophrenia patients, compared to controls [116]. A study by Zhu et al. [101] revealed that transplantation of fecal microbiota from schizophrenia patients into antibiotic-treated mice resulted in schizophrenia-like behaviors as well as reduced tryptophan and increased levels of dopamine in the PFC and serotonin in the hippocampus.

There is also evidence to demonstrate that several antipsychotics have antibiotic properties and can affect the composition of the gut microbiome [118,119]. The gut microbiome has also been identified as a factor mediating metabolic side effects of second-generation antipsychotics [120,121]. The chronic use of antipsychotics can cause dysbiosis of the gut microbiome, with resulting dysregulation of the neurotransmitters dopamine, glutamate, serotonin, and noradrenaline; increases in inflammation pathways; oxidative stress; and mitochondrial dysfunction [119]. The consequences may include resistance to treatment and increased severity of metabolic irregularities [122].

## 6. Oxidative Stress

Oxidative stress is often described as the result of an imbalance between the production of reactive species, or free radicals, and the inability of the body to detoxify these reactive products [57,123]. The excess production of these reactive species can result in molecular damage, cellular dysfunction, neurotoxicity, and activation of both apoptotic and necrotic cell-death pathways [8]. Most of the literature reports on oxidative stress deal with reactive oxygen species (ROS) (e.g., the superoxide free radical and hydrogen peroxide), but there can also be reactive nitrogen species (RNS) such as the nitroxyl anion and various nitrogen oxides. ROS are produced as by-products of the mitochondrial production of adenosine triphosphate (ATP) [123]. Under healthy conditions, the body can maintain a balance between oxidation and reduction in tissues (redox balance). In conditions of high stress, free radicals may have a negative effect on molecules such as lipids, proteins, and nucleic acids. The body has a set of enzymatic (e.g., superoxide dismutase, catalase, and glutathione peroxidase) and non-enzymatic (e.g., glutathione, metal binding proteins, and uric acid) antioxidant defenses to combat the excessive accumulation of ROS and RNS [123]. It has been proposed that maternal infection during pregnancy may be a risk factor for the development of disorders such as such as schizophrenia in the offspring [124]. In a study in pregnant rats treated with the bacterial endotoxin lipopolysaccharide (LPS) to mimic maternal infection, Lante et al. [124] investigated biochemical changes in the hippocampi of the fetuses. Oxidative stress, as shown by a rapid increase in protein carbonylation and a decrease in the levels of α-tocopherol and glutathione, was observed in the male, but not the female, fetuses. Similarly, impairment of NMDAR synaptic currents, long-term potentiation in the CA1 region, and spatial recognition in the water maze test were observed in male, but not female, 28-day-old offspring. These LPS-induced changes in the male fetuses and 28-day-old males were prevented by pretreatment with the antioxidant N-acetylcysteine (NAC) [124]. This study also highlights the importance of designing studies which include both sexes.

ROS production is an inherent part of aging and is influenced by environmental and genetic factors. However, in pathological conditions such as psychosis, oxidative stress exacerbates and accelerates the extent of neuronal damage through mitochondrial dysfunction and pro-apoptotic pathways. Oxidative stress bidirectionally communicates with multiple physiological mechanisms, extending its effects to neurotransmission, neuroinflammation and homeostatic networks such as the HPA axis. Murray et al. [123] have described how oxidative stress may be involved in hypotheses of psychosis involving dopamine, glutamate, the immune system, and disrupted brain connectivity. Fraguas et al. [125] conducted a systematic review of cross-sectional studies comparing blood markers of inflammation and oxidative stress in first-episode psychosis (FEP) patients and healthy controls. They found lower total antioxidant status and docosahexaenoic acid levels and higher levels of homocysteine, IL-6, and TNF-α in FEP patients, compared to controls. It was concluded that there was reduced antioxidant status and a pro-inflammatory imbalance in FEP patients. 

Oxidative stress seems to be particularly important with regard to dopamine. ROS can block the dopamine transporters (DATs) which regulate dopamine degradation [126]. Rodent models demonstrated that these effects are present in the mesolimbic pathway, as increased dopamine concentrations are observed in the synaptic cleft in the nucleus accumbens following decreased reuptake [126]. A major source of oxidative stress in the brain is thought to originate from the auto-oxidation of increased dopamine levels [127,128]. Dopamine also decreases the activity of antioxidant systems, augmenting the effects of oxidative stress [126]. Grima et al. [129] studied cultured cortical neurons with a low concentration of glutathione in the medium and found that dopamine decreased that concentration by 40%. They proposed that this effect was the result of conjugation of a semiquinone/quinone metabolite of dopamine with glutathione. 

Jitca et al. [126] reported that administering psychostimulants that increase dopamine concentrations in the synaptic cleft exerts a pro-oxidative effect in localized areas implicated in psychosis, such as the hippocampus and PFC. Positron emission tomography (PET) imaging has indicated that increased dopaminergic neurotransmission can be neurotoxic, leading to increased production of ROS [130]. Additionally, psychostimulants may decrease the capacity of protective antioxidant enzymes [126]. 

As previously stated, NMDAR hypofunction and increased release of glutamate are characteristics of psychosis. The resultant glutamate-induced excitotoxicity is associated with the production of ROS and oxidative stress [126]. Mikulska et al. [92] also noted a correlation between oxidative stress and the symptoms of patients with first-episode psychosis. There is an evident bidirectional relationship between oxidative stress and NMDA hypofunction. On the one hand, NMDAR hypofunction could lead to uncontrolled oxidation and high neurotoxicity in parvalbumin interneurons (PVIs) [57]. PVIs are inhibitory GABAergic neurons that are partly responsible for maintaining an E/I balance [57]. These neurons have critical functions in neurochemical communication, and they have a high metabolic demand, making them increasingly susceptible to oxidative stress and severe damage [57]. Both immune dysfunction and oxidative stress lead to the pruning of PVIs, which is thought to be a key pathophysiology involved in psychosis [51]. Preclinical studies on rat models have also suggested that impaired function of PVIs leads to elevated dopamine in the mesolimbic system, contributing to the hyperactivity of dopamine [130]. As oxidative stress precedes deficits in PVIs, NMDAR hypofunction can be viewed as a secondary consequence of oxidative stress [57]. Amphetamine-induced neurotoxicity following oxidative stress has been proposed as being mediated by NMDAR glutamatergic neurotransmission [126]. Both NMDAR hypofunction and PVI disruption are hallmarks of psychosis [57]. 

Reactive astrocytes and microglia are capable of producing ROS, and not only induce increased pro-inflammatory cytokine release, chronic inflammation, and cell death, but are capable of perpetuating a reinforcing cycle [57]. Thus, immune activity, which is elevated in psychosis, is considered a major source of oxidative stress. On the other hand, oxidative stress can induce neuroinflammation, demonstrating the bidirectional mechanism of action underlying psychosis. Some studies suggest that psychosis in immune activation models is mediated by increased oxidative stress [57]. 

Reactive microglia are also found to disrupt BBB function through the increased secretion of pro-inflammatory cytokines and ROS [54]. Oligodendrocytes and their precursor cells are also notably susceptible to oxidative stress, in which ROS disrupts physiological signal transduction and the formation of myelin [33]. The finding that oxidative stress and the neuroinflammatory processes of the immune system interact in pathophysiological conditions suggests that oxidative stress may be both the cause and consequence of neuroinflammation [131].

There is also clinical evidence to support the claim that oxidative stress is an underlying neurobiological mechanism of psychosis, rather than a secondary consequence of pathophysiology [38,123,128]. In a comprehensive review, Ermakov et al. [38] stated that there is overwhelming evidence of redox imbalance in schizophrenia and described several factors that contribute to a pro-oxidant state and redox imbalance. At the molecular level, the mechanisms involved include genetic and environmental factors that increase susceptibility to oxidative stress and result in altered redox signalling related to glutathione deficiency and impaired function of redox-sensitive transcriptional factors (e.g., Nrf2, FoxO, and NF-kB). The results at the cellular level may include mitochondrial dysfunction, abnormal myelination, NMDAR hypofunction, and dysfunction of PVIs [38,123]. Murray et al. [123] have stated that oxidative stress is probably not the cause of schizophrenia but may be part of the reason for the declining function and poor outcomes. These authors have commented on the close association between the antioxidant glutathione and NMDAR activity. A decrease in the levels of glutathione, as has been reported in schizophrenia patients, results in NMDAR hypofunction [132,133]. In a comprehensive review of blood oxidative stress markers, Rambaud et al. [128] reported increased oxidative damage to lipids and decreased levels of non-enzymatic antioxidants in the blood of schizophrenia patients. 

Therapeutic interventions that target ROS have also been explored. Jitca et al. [126] discussed the application of antioxidant therapies such as ascorbic acid (Vitamin C), since it reduces the production of ROS and limits cell damage through regulation of molecules involved in apoptosis. Exogenous administration of melatonin is also protective against oxidative stress where an underlying mechanism of melatonin is regulation of glutamate levels and concentrations of pro-inflammatory cytokines [126]. Similarly, Xu and Fang [8] reported that administering the antioxidant NAC improves cognitive psychotic symptoms in schizophrenia. From the clinical studies conducted, Xu and Fang [8] concluded that a combined therapy which involved the administration of antioxidants in conjunction with antipsychotics is a promising avenue of treatment for psychotic disorders. The experimental administration of minocycline, an antibiotic with both anti-inflammatory and antioxidant properties, improved cognitive deficits induced by the chronic administration of PCP in animal models [59]. However, it should also be mentioned that there is also some disagreement in the literature about the effectiveness of antioxidant treatments added to antipsychotics in order to improve symptoms in schizophrenia [123,134,135].

## 7. Mitochondrial Dysfunction

Mitochondria, which play crucial roles in energy metabolism, oxidative stress, and modulation of synaptic activity, produce almost all of the cellular ATP in the body, via oxidative phosphorylation carried out by complexes I through IV of the electron transport chain [136,137]. As pointed out by Roberts [138] in a comprehensive review of post-mortem studies in schizophrenia, conducting studies on mitochondrial dysfunction is very difficult. In schizophrenia there are differential effects depending on the brain region, cellular and subcellular location of the mitochondria, predominant symptoms, and the treatment involved. There have been deficits in the expressions of multiple genes related to mitochondrial function reported in many schizophrenia cohorts (see tables in [138]). Hjelm et al. [139], in a review of the literature in this area, identified 57 mitochondrial genes reported to be dysregulated (mainly downregulated) in at least two independent studies. Prominent findings in schizophrenia have included decreased activity of complexes I and IV and abnormal levels of individual subunits that make up the electron transport chain complexes [138]. Depending on the brain region and the type of cells studied (e.g., neurons, astrocytes, and oligodendrocytes), there may be changes in the number or size of the mitochondria [138].

Research to date strongly suggests that interactions among mitochondrial deficits, neurotransmitter dysfunction, oxidative stress, and inflammation produce the symptoms of schizophrenia [140]. Prabakaran et al. [141] conducted a comprehensive study utilizing transcriptomics, proteomics, and metabolomics on postmortem human brain PFC tissue from subjects with schizophrenia and matched controls. These researchers found numerous differences between the two groups, with nearly half of the altered proteins identified by proteomics being associated with responses related to mitochondrial function and oxidative stress. They hypothesized that the acute and chronic deficits seen in schizophrenia are the results of diverse genetic and/or epigenetic factors producing hypoxic events in a constitutively vulnerable PFC. There have been reports for many years of abnormal energy generation in schizophrenia, accompanied by increased lactate levels and decreased glutathione levels in the brain and CSF [142,143,144]. Dopamine can produce deleterious effects in psychosis through multiple processes such as inflammation, oxidative stress, and mitochondrial impairment [145,146]. Studies in vitro on neurons showed that dopamine can lead to apoptosis through mitochondrial dysfunction, reduce cellular ATP, and cause impairment of mitochondria via inhibition of complex I [147]. With regard to interactions with glutamate, the excessive Ca^2+^ influx through NMDARs found in schizophrenia can cause calcium overload of mitochondria and subsequent potential mitochondrial dysfunction and activation of cell-death signals [140,148]. There is also evidence for bidirectional interactions between mitochondrial dysfunction and oxidative stress and inflammation. Oxidative/nitrosative stress has been reported in schizophrenia [149,150], where it can cause impairments of mitochondrial oxidative phosphorylation [151,152]. Under normal conditions, mitochondria have an antioxidant defense system, but alterations in that defense system have been reported in schizophrenia [153,154,155]. Dysfunction of the mitochondrial complex could also be a source of oxidative stress in schizophrenia [156]. With regard to inflammation, ROS produced by mitochondria can upregulate the production of pro-inflammatory cytokines [157]. Conversely, increased levels of pro-inflammatory cytokines may perturb the mitochondrial anti-oxidative defense system in schizophrenia patients [156,158].

## 8. Discussion, Conclusions and Future Directions

This paper has reviewed several important facets of interest in psychosis, namely neurotransmitter dysregulation, neuroinflammation, oxidative stress, and mitochondrial dysfunction, which are implicated in psychosis pathogenesis.

The dopamine hypothesis was crucially important for expanding knowledge of the neurobiology of psychosis and developing antipsychotic drugs. However, it was obvious that psychosis was multifaceted, and that other systems in addition to the dopaminergic system were involved in the etiology of the symptoms. There was considerable interest in serotonin as a possible target for antipsychotics, and in recent decades there has been an enormous amount of research conducted that has provided evidence of the hypoactivity of NMDARs and the subsequent release of glutamate in psychosis. Considerable research has indicated interactions among dopamine, glutamate, GABA, and serotonin, perturbations of which may contribute to symptoms of psychosis.

In order to fully understand the etiology of psychosis and develop drug regimens with wider applicability and reduced side effects, it was necessary to expand research beyond neurotransmitter dysregulation and to understand other factors that may contribute to the symptoms of psychosis. There has been considerable research performed on the role of the endocrine system, primarily involving the HPA axis, and as to the immune system in psychiatric disorders in general. As indicated in the current review, dysfunction of these systems is also evident in some people suffering from psychosis. This interest in the immune system has led to extensive research on microglia because of their involvement in inflammation and cytokine release. Additional research has also demonstrated interesting effects of other glia, i.e., astrocytes and oligodendrocytes, which may contribute to psychosis. With both types of glia there are interactions with the immune system, and BBB hyperpermeability and abnormalities in myelination have been reported in psychosis, with the former apparently related primarily to astrocyte dysfunction and the latter to oligodendrocytes.

Oxidative stress has been reported to be associated with a variety of psychiatric and neurological disorders, including psychosis. There is now extensive research demonstrating that oxidative stress can be a product of neurotransmitter dysfunction (e.g., hyperactivity of dopamine) and dysfunctions of glial cells, the endocrine system, and mitochondria. In recent years, research activity on the GBA and its involvement in the etiology and pharmacotherapy of neurological and psychiatric disorders has intensified. It has now been well documented that this axis interacts bidirectionally with the immune and endocrine systems. Since there is often low-level inflammation associated with psychosis, activity in the GBA is likely a contributing factor. Mitochondrial dysfunction obviously contributes to oxidative stress, but there is also an interaction of mitochondrial dysfunction with neurotransmitter function and the immune system in psychosis. Abnormal energy generation has been reported for many years in schizophrenia, and the mitochondria are primarily responsible for ATP production and energy generation. Deficits in numerous genes related to mitochondrial function have been reported in psychosis in many studies.

In this review paper, we have focused on the factors mentioned in the above sections, but we realize that there is an enormous amount of literature on the role of genetics in psychotic disorders, particularly schizophrenia. It has been proposed that schizophrenia results from interactions between genetic predisposition and environmental influences (e.g., birth complications, stress, toxins, and drugs ([33] for review)), with regulation by epigenetic changes [33,159]. There have been extensive studies showing that epigenetic changes in schizophrenia contribute to the hypofunction of NMDARs [160], regulation of dopaminergic and GABAergic activity [161], and regulation of immune and neuroinflammatory responses through the alteration of expression of genes for proinflammatory cytokines [162]. It has been proposed that prenatal stress-induced epigenetic changes in the reelin gene may be related to neurodevelopmental changes observed in the PFC in schizophrenia [163,164]. Epigenetic changes may also be related to the oligodendrocyte abnormalities in schizophrenia, since they can alter the transcriptions of genes involved in differentiation of oligodendrocytes and myelination [165]. In a comprehensive review, Fisar [33] discusses a neurodevelopment–vulnerability–neurochemical model in which the impairment of synaptic plasticity and neurotransmission in certain neuronal circuits in the brain is affected by genetic and epigenetic factors and environmental influences. The result of impaired neurodevelopment is increased sensitivity to stress and/or inflammation [33]. Regarding the pharmacotherapy of schizophrenia, antipsychotics have been proposed to affect epigenetic regulation, although further research is necessary to fully understand the significance of these effects [164]. Epigenetic drugs such as histone demethylase inhibitors, histone deacetylase inhibitors and DNA methyltransferase inhibitors are being investigated as drugs possibly to be included in the treatment of schizophrenia [166,167].

There has also been a great deal of research performed identifying possible biomarkers in psychotic disorders. We have included information on biomarkers in most of the sections described above in this review, but a detailed overview of the enormous number of biomarkers that have been investigated is beyond the scope of this review. There are numerous excellent papers on this topic in the literature (e.g., [33,168,169,170,171,172,173]). The Yue et al. [172] paper contains detailed tables on potential diagnostic biomarkers in the following categories: neuroimmune markers [CNS and peripheral cytokines and C-reactive protein (CRP)]; neurotransmitter-related metabolic markers; fatty acids; neuroactive steroids; neurotrophins; neuroimaging; genetics; and electrophysiology. Yu et al. [173] present results from a study on plasma levels of neuropeptides in patients with first-episode schizophrenia (FES), bipolar disorder (BD), or major depressive disorder and healthy controls. Levels of neurotensin, α-melanocyte-stimulating hormone (α-MSH), orexin-A, oxytocin, and substance P were decreased in all three patient groups compared to the levels in the healthy controls but did not provide a significant differentiation between the three patient groups. Across the entire sample, neurotensin was associated with better executive function. In the FES and BD groups, there was an association of increased psychotic symptoms with lower levels of oxytocin and higher levels of substance P [173].

The exciting and innovative research that has been performed on psychosis in the past few decades has greatly increased our knowledge of factors involved in the etiology of psychosis, but has also made us acutely aware of how complex psychotic disorders are. Although the research has indicated some new areas to pursue as possible drug targets, there have not been recent big breakthroughs in new drug regimens for treating psychosis. Part of the problem is that many of the possible biomarkers in psychotic disorders are also present in other neuropsychiatric disorders. Clinical heterogeneity of schizophrenia is a complicating factor [174]. There is also always the concern that peripheral biomarkers may not reflect what is happening in the CNS [168]. In addition, co-occurrence of other disorders in psychotic disorders (co-morbidity) is common, confounding diagnosis and treatment. Many of the researchers cited in the current review paper have indicated in their papers that future studies on psychosis should involve more longitudinal investigations that take into account factors such as number and sex of subjects, presence of drug treatments, lifestyle and other risk factors, co-occurrence of other disorders, age of onset, prevalence of positive and negative symptoms, and nutritional status [71,168,175,176]. Where possible, approaches using multiple techniques should be used and the findings should be related to specific symptoms [104,167,168,172,177]. It is very important to compare biomarkers over the clinical course of schizophrenia [45,119,166]. Many preclinical and clinical studies have been conducted on the GBA in recent years. The findings have been exciting, but there is considerable inter-individual variation in the fecal microbial findings (although this may be useful for future precision health-based diagnosis and treatment). More extensive longitudinal studies on prebiotics and probiotics need to be conducted before they can be considered as routine compositions to be given in conjunction with antipsychotics. Such studies should be combined with metabolomic investigations and consider factors such as nutritional status, sex, and stage of the illness [96,117,119,121,178]. There have been increasing studies on connectomics, neuroimaging, and the use of artificial intelligence and machine learning in investigations on psychosis in recent years, and these will no doubt continue to expand in the future.

The wide-ranging, interactive mechanisms which apparently underlie psychosis can be viewed as part of dysfunction of an overarching regulatory network that essentially integrates multiple physiological mechanisms. Despite the challenges posed by studying psychiatric symptoms, progress in the understanding of the pathophysiology of psychosis is of interest to further spur on the development of new diagnostic procedures and personalized interventions. The research to date has revealed several new possible targets for treatment, which is crucial, since drug development in psychosis treatment has been relatively discouraging. Although the drugs currently available have been of enormous aid to many patients, many other patients do not respond well, and the drugs can have serious adverse effects. Contemporary knowledge about the neurobiological mechanisms involved in psychosis reinforces the understanding that the etiology of psychosis is multifactorial, emphasizing the need for multidisciplinary approaches to the management of psychosis. Further understanding of the neuroendocrine, immunomodulatory, and neurobiological mechanisms within psychosis is of relevance in the field of psychiatry, as it is very important for seeking pharmacological, psychiatric, and psychological interventions that may be protective, reduce susceptibility, and limit the progression of damage, or even reverse both the physiological and behaviorally induced damage.

## Figures and Tables

**Table 1:** Some properties of glia which have potential relevance to psychosis. Information in this table is taken from the material referenced in the text of this review. ROS = reactive oxygen species; RNS = reactive nitrogen species; BBB = blood–brain barrier.

Microglia	Astrocytes	Oligodendrocytes
When activated, can cause inflammation by release of cytokines and may be involved in excessive synaptic pruning, loss of cortical volume, and cellular dysfunction in the PFC Reactivity can be increased by NMDAR antagonists Activation is reduced by some antipsychotics May disrupt the BBB through secretion of ROS and cytokines	Express a wide variety of receptors, transporter enzymes, and ion channels Regulate nutrition of neurons, formation and modulation of synapses, and BBB function Regulate glutamate and GABA transport Involved in secretion of synaptogenic and neurotrophic factors, but can also eliminate synapses Important in the glutamate–glutamine cycle Can influence synthesis of D-serine Regulation of oxidative stress and inflammation Can be a source of ROS and RNS related to mitochondrial dysfunction Conversely, oxidative stress can produce adverse effects on astrocytes	Myelinating cells of the CNS Density reported to be lowered in schizophrenia The NMDAR antagonist PCP leads to impaired regulation of myelination and reduced levels of oligodendrocytes in mouse frontal cortex Loss of oligodendrocytes may result in infiltration of proinflammatory cytokines and reactive microglia They and their precursor cells are susceptible to oxidative stress There is cross-talk among microglia, astrocytes and oligodendrocytes

## List of Abbreviations

BBB	Blood–brain barrier
BD	Bipolar disorder
B-GOS	Galacto-oligosaccharide
CHR	Clinical high risk
CNS	Central nervous system
CRP	C-Reactive protein
CSF	Cerebrospinal fluid
DAT	Dopamine transporter
DISC	Disrupted-in-schizophrenia 1
DSM-5-TR	Diagnostic and Statistical Manual of Mental Disorders, 5th edition, Text Revision
FEP	First-episode psychosis
FES	First-episode schizophrenia
GAT	GABA transporter
GABA	γ-Aminobutyric acid
GBA	Gut–brain axis
GF	Germ-free
GI	Gastrointestinal
HPA Axis	Hypothalamic–pituitary–adrenal axis
5-HT	5-Hydroxytryptamine, Serotonin
INF	Interferon
IL	Interleukin
IP_3_	Inositol 1,4,5-triphosphate
LSD	Lysergic acid diethylamide
LPS	Lipopolysaccharide
MIA	Maternal immune activation
MK-801	Dizocilpine
α-MSH	α-Melanocyte-stimulating hormone
NAC	N-Acetylcysteine
NMDA	N-Methyl-D-aspartate
NMDAR	N-Methyl-D-aspartate glutamate receptor
PVIs	Parvalbumin interneurons
PCP	Phencyclidine
PET	Positron emission tomography
PFC	Prefrontal cortex
RNS	Reactive nitrogen species
ROS	Reactive oxygen species
SCFA	Short chain fatty acid
TGF-β1	Transforming growth factor-β1
TNF	Tumor necrosis factor
VTA	Ventral tegmental area

## References

[1] Gaebel W., Zielasek J. (2015). Focus on Psychosis. Dialogues Clin. Neurosci..

[2] Calabrese J., Al Khalili Y. (2024). Psychosis. StatsPearls [Internet].

[3] American Psychiatric Association (2013). Diagnostic and Statistical Manual of Mental Disorders.

[4] Stahl S.M. (2018). Beyond the Dopamine Hypothesis of Schizophrenia to Three Neural Networks of Psychosis: Dopamine, Serotonin, and Glutamate. CNS Spectr..

[5] van Rossum J.M. (1966). The Significance of Dopamine-Receptor Blockade for the Mechanism of Action of Neuroleptic Drugs. Arch. Int. Pharmacodyn. Ther..

[6] Seeman P. (1987). Dopamine Receptors and the Dopamine Hypothesis of Schizophrenia. Synapse.

[7] Carlsson M.L., Carlsson A., Nilsson M. (2004). Schizophrenia: From Dopamine to Glutamate and Back. Curr. Med. Chem..

[8] Xu H., Yang F. (2022). The Interplay of Dopamine Metabolism Abnormalities and Mitochondrial Defects in the Pathogenesis of Schizophrenia. Transl. Psychiatry.

[9] Kesby J.P., Eyles D.W., McGrath J.J., Scott J.G. (2018). Dopamine, Psychosis and Schizophrenia: The Widening Gap between Basic and Clinical Neuroscience. Transl. Psychiatry.

[10] Hsu T.M., McCutcheon J.E., Roitman M.F. (2018). Parallels and Overlap: The Integration of Homeostatic Signals by Mesolimbic Dopamine Neurons. Front. Psychiatry.

[11] Davis K.L., Kahn R.S., Ko G., Davidson M. (1991). Dopamine in Schizophrenia: A Review and Reconceptualization. Am. J. Psychiatry.

[12] Howes O.D., Kapur S. (2009). The Dopamine Hypothesis of Schizophrenia: Version III--the Final Common Pathway. Schizophr. Bull..

[13] Buck S.A., Quincy Erickson-Oberg M., Logan R.W., Freyberg Z. (2022). Relevance of Interactions between Dopamine and Glutamate Neurotransmission in Schizophrenia. Mol. Psychiatry.

[14] Thompson I.A., De Vries E.F.J., Sommer I.E.C. (2020). Dopamine D2 Up-Regulation in Psychosis Patients after Antipsychotic Drug Treatment. Curr. Opin. Psychiatry.

[15] Murray R.M., Englund A., Abi-Dargham A., Lewis D.A., Di Forti M., Davies C., Sherif M., McGuire P., D’Souza D.C. (2017). Cannabis-Associated Psychosis: Neural Substrate and Clinical Impact. Neuropharmacology.

[16] Abi-Dargham A., Gil R., Krystal J., Baldwin R.M., Seibyl J.P., Bowers M., van Dyck C.H., Charney D.S., Innis R.B., Laruelle M. (1998). Increased Striatal Dopamine Transmission in Schizophrenia: Confirmation in a Second Cohort. Am. J. Psychiatry.

[17] Hamilton I., Monaghan M. (2019). Cannabis and Psychosis: Are We Any Closer to Understanding the Relationship?. Curr. Psychiatry Rep..

[18] Aryutova K., Stoyanov D. (2021). Pharmaco-Magnetic Resonance as a Tool for Monitoring the Medication-Related Effects in the Brain May Provide Potential Biomarkers for Psychotic Disorders. Int. J. Mol. Sci..

[19] Kesby J.P., Turner K.M. (2024). The Translational Utility of Circuit-Based Manipulations in Preclinical Models. Biol. Psychiatry Glob. Open Sci..

[20] Tranter M.M., Faget L., Hnasko T.S., Powell S.B., Dillon D.G., Barnes S.A. (2024). Postnatal Phencyclidine-Induced Deficits in Decision Making Are Ameliorated by Optogenetic Inhibition of Ventromedial Orbitofrontal Cortical Glutamate Neurons. Biol. Psychiatry Glob. Open Sci..

[21] Moghaddam B., Javitt D. (2012). From Revolution to Evolution: The Glutamate Hypothesis of Schizophrenia and Its Implication for Treatment. Neuropsychopharmacology.

[22] Mei Y.-Y., Wu D.C., Zhou N. (2018). Astrocytic Regulation of Glutamate Transmission in Schizophrenia. Front. Psychiatry.

[23] Shin E.J., Dang D.K., Tran T.V., Tran H.Q., Jeong J.H., Nah S.Y., Jang C.G., Yamada K., Nabeshima T., Kim H.C. (2017). Current Understanding of Methamphetamine-Associated Dopaminergic Neurodegeneration and Psychotoxic Behaviors. Arch. Pharm. Res..

[24] Oda Y., Fujita Y., Oishi K., Nakata Y., Takase M., Niitsu T., Kanahara N., Shirayama Y., Hashimoto K., Iyo M. (2017). Alterations in Glutamatergic Signaling in the Brain of Dopamine Supersensitivity Psychosis and Non-Supersensitivity Psychosis Model Rats. Psychopharmacology.

[25] Beck K., Hindley G., Borgan F., Ginestet C., McCutcheon R., Brugger S., Driesen N., Ranganathan M., D’Souza D.C., Taylor M. (2020). Association of Ketamine with Psychiatric Symptoms and Implications for Its Therapeutic Use and for Understanding Schizophrenia. JAMA Netw. Open.

[26] Ranson A., Broom E., Powell A., Chen F., Major G., Hall J. (2019). Top-Down Suppression of Sensory Cortex in an NMDAR Hypofunction Model of Psychosis. Schizophr. Bull..

[27] Oranje B., Gispen-de Wied C.C., Westenberg H.G.M., Kemner C., Verbaten M.N., Kahn R.S. (2009). Haloperidol Counteracts the Ketamine-Induced Disruption of Processing Negativity, but Not That of the P300 Amplitude. Int. J. Neuropsychopharmacol..

[28] Malhotra A.K., Adler C.M., Kennison S.D., Elman I., Pickar D., Breier A. (1997). Clozapine Blunts N-Methyl-d-Aspartate Antagonist-Induced Psychosis: A Study with Ketamine. Biol. Psychiatry.

[29] Howes O., McCutcheon R., Stone J. (2015). Glutamate and Dopamine in Schizophrenia: An Update for the 21st Century. J. Psychopharmacol..

[30] Labrie V., Wong A.H.C., Roder J.C. (2012). Contributions of the D-Serine Pathway to Schizophrenia. Neuropharmacology.

[31] Nishikawa T., Umino A., Umino M., Riederer P., Laux G., Nagatsu T., Le W., Riederer C. (2022). D-Serine: Basic Aspects with a Focus on Psychosis. NeuroPsychopharmacotherapy.

[32] Nishikawa T., Umino A., Umino M., Riederer P., Laux G., Nagatsu T., Le W., Riederer C. (2022). D-Serine in the Treatment of Psychosis. NeuroPsychopharmacotherapy.

[33] Fišar Z. (2023). Biological Hypotheses, Risk Factors, and Biomarkers of Schizophrenia. Prog. Neuropsychopharmacol. Biol. Psychiatry.

[34] Eggers A.E. (2013). A Serotonin Hypothesis of Schizophrenia. Med. Hypotheses.

[35] Chiang M., Lombardi D., Du J., Makrum U., Sitthichai R., Harrington A., Shukair N., Zhao M., Fan X. (2019). Methamphetamine-Associated Psychosis: Clinical Presentation, Biological Basis, and Treatment Options. Hum. Psychopharmacol..

[36] González-Maeso J., Ang R.L., Yuen T., Chan P., Weisstaub N.V., López-Giménez J.F., Zhou M., Okawa Y., Callado L.F., Milligan G. (2008). Identification of a Serotonin/Glutamate Receptor Complex Implicated in Psychosis. Nature.

[37] Murphy C.E., Walker A.K., Weickert C.S. (2021). Neuroinflammation in Schizophrenia: The Role of Nuclear Factor Kappa B. Transl. Psychiatry.

[38] Ermakov E.A., Dmitrieva E.M., Parshukova D.A., Kazantseva D.V., Vasilieva A.R., Smirnova L.P. (2021). Oxidative Stress-Related Mechanisms in Schizophrenia Pathogenesis and New Treatment Perspectives. Oxid. Med. Cell Longev..

[39] Ansari Z., Pawar S., Seetharaman R. (2022). Neuroinflammation and Oxidative Stress in Schizophrenia: Are These Opportunities for Repurposing?. Postgrad. Med..

[40] Comer A.L., Carrier M., Tremblay M.È., Cruz-Martín A. (2020). The Inflamed Brain in Schizophrenia: The Convergence of Genetic and Environmental Risk Factors That Lead to Uncontrolled Neuroinflammation. Front. Cell Neurosci..

[41] Upthegrove R., Khandaker G.M., Khandaker G.M., Meyer U., Jones P.B. (2020). Cytokines, Oxidative Stress, and Cellular Markers of Inflammation in Schizophrenia. Neuroinflammation and Schizophrenia.

[42] Mondelli V., Blackman G., Kempton M.J., Pollak T.A., Iyegbe C., Valmaggia L.R., Amminger P., Barrantes-Vidal N., Bressan R., van der Gaag M. (2023). Serum Immune Markers and Transition to Psychosis in Individuals at Clinical High Risk. Brain Behav. Immun..

[43] Kim H., Baek S.-H., Kim J.-W., Ryu S., Lee J.-Y., Kim J.-M., Chung Y.-C., Kim S.-W. (2023). Inflammatory Markers of Symptomatic Remission at 6 Months in Patients with First-Episode Schizophrenia. Schizophrenia.

[44] Yang X., Wang Y., Li Q., Zhong Y., Chen L., Du Y., He J., Liao L., Xiong K., Yi C. (2018). The Main Molecular Mechanisms Underlying Methamphetamine- Induced Neurotoxicity and Implications for Pharmacological Treatment. Front. Mol. Neurosci..

[45] Miller B.J., Goldsmith D.R. (2017). Towards an Immunophenotype of Schizophrenia: Progress, Potential Mechanisms, and Future Directions. Neuropsychopharmacology.

[46] Hansen N., Malchow B. (2023). Monoclonal Antibody Therapy in Autoantibody-Associated Psychotic Disorders and Schizophrenia: Narrative Reviews of Past and Current Clinical Trials. Psychiatr. Danub..

[47] Weickert T.W., Jacomb I., Lenroot R., Lappin J., Weinberg D., Brooks W.S., Brown D., Pellen D., Kindler J., Mohan A. (2024). Adjunctive Canakinumab Reduces Peripheral Inflammation Markers and Improves Positive Symptoms in People with Schizophrenia and Inflammation: A Randomized Control Trial. Brain Behav. Immun..

[48] Reale M., Costantini E., Greig N.H. (2021). Cytokine Imbalance in Schizophrenia. From Research to Clinic: Potential Implications for Treatment. Front. Psychiatry.

[49] de Bartolomeis A., Barone A., Vellucci L., Mazza B., Austin M.C., Iasevoli F., Ciccarelli M. (2022). Linking Inflammation, Aberrant Glutamate-Dopamine Interaction, and Post-Synaptic Changes: Translational Relevance for Schizophrenia and Antipsychotic Treatment: A Systematic Review. Mol. Neurobiol..

[50] Ellman L.M., Deicken R.F., Vinogradov S., Kremen W.S., Poole J.H., Kern D.M., Tsai W.Y., Schaefer C.A., Brown A.S. (2010). Structural Brain Alterations in Schizophrenia Following Fetal Exposure to the Inflammatory Cytokine Interleukin-8. Schizophr. Res..

[51] Kabiersch A., Furukawa H., Del Rey A., Besedovsky H.O. (1998). Administration of Interleukin-1 at Birth Affects Dopaminergic Neurons in Adult Mice ^a^. Ann. N. Y Acad. Sci..

[52] Felger J.C., Mun J., Kimmel H.L., Nye J.A., Drake D.F., Hernandez C.R., Freeman A.A., Rye D.B., Goodman M.M., Howell L.L. (2013). Chronic Interferon-α Decreases Dopamine 2 Receptor Binding and Striatal Dopamine Release in Association with Anhedonia-Like Behavior in Nonhuman Primates. Neuropsychopharmacology.

[53] Feng Y., Lu Y. (2021). Immunomodulatory Effects of Dopamine in Inflammatory Diseases. Front. Immunol..

[54] Pollak T.A., Drndarski S., Stone J.M., David A.S., McGuire P., Abbott N.J. (2018). The Blood–Brain Barrier in Psychosis. Lancet Psychiatry.

[55] Wohleb E.S. (2016). Neuron-Microglia Interactions in Mental Health Disorders: “For Better, and For Worse”. Front. Immunol..

[56] Bloomfield P.S., Selvaraj S., Veronese M., Rizzo G., Bertoldo A., Owen D.R., Bloomfield M.A.P., Bonoldi I., Kalk N., Turkheimer F. (2016). Microglial Activity in People at Ultra High Risk of Psychosis and in Schizophrenia: An [11C]PBR28 PET Brain Imaging Study. Am. J. Psychiatry.

[57] Barron H., Hafizi S., Andreazza A.C., Mizrahi R. (2017). Neuroinflammation and Oxidative Stress in Psychosis and Psychosis Risk. Int. J. Mol. Sci..

[58] Parellada E., Gassó P. (2021). Glutamate and Microglia Activation as a Driver of Dendritic Apoptosis: A Core Pathophysiological Mechanism to Understand Schizophrenia. Transl. Psychiatry.

[59] Mattei D., Schweibold R., Wolf S.A. (2015). Brain in Flames—Animal Models of Psychosis: Utility and Limitations. Neuropsychiatr. Dis. Treat..

[60] Czapski G.A., Strosznajder J.B. (2021). Glutamate and GABA in Microglia-Neuron Cross-Talk in Alzheimer’s Disease. Int. J. Mol. Sci..

[61] Notter T. (2021). Astrocytes in Schizophrenia. Brain Neurosci. Adv..

[62] Fiebig C., Keiner S., Ebert B., Schäffner I., Jagasia R., Lie D.C., Beckervordersandforth R. (2019). Mitochondrial Dysfunction in Astrocytes Impairs the Generation of Reactive Astrocytes and Enhances Neuronal Cell Death in the Cortex upon Photothrombotic Lesion. Front. Mol. Neurosci..

[63] Chen Y., Qin C., Huang J., Tang X., Liu C., Huang K., Xu J., Guo G., Tong A., Zhou L. (2020). The Role of Astrocytes in Oxidative Stress of Central Nervous System: A Mixed Blessing. Cell Prolif..

[64] Schiera G., Di Liegro C.M., Schirò G., Sorbello G., Di Liegro I. (2024). Involvement of Astrocytes in the Formation, Maintenance, and Function of the Blood–Brain Barrier. Cells.

[65] Rothstein J.D., Dykes-Hoberg M., Pardo C.A., Bristol L.A., Jin L., Kuncl R.W., Kanai Y., Hediger M.A., Wang Y., Schielke J.P. (1996). Knockout of Glutamate Transporters Reveals a Major Role for Astroglial Transport in Excitotoxicity and Clearance of Glutamate. Neuron.

[66] Purushotham S.S., Buskila Y. (2023). Astrocytic Modulation of Neuronal Signalling. Front. Network Physiol..

[67] Sonnewald U., Schousboe A. (2016). Introduction to the Glutamate–Glutamine Cycle. Adv. Neurobiol..

[68] Dietz A.G., Goldman S.A., Nedergaard M. (2020). Glial Cells in Schizophrenia: A Unified Hypothesis. Lancet Psychiatry.

[69] Hashimoto K., Engberg G., Shimizu E., Nordin C., Lindström L.H., Iyo M. (2005). Elevated Glutamine/Glutamate Ratio in Cerebrospinal Fluid of First Episode and Drug Naive Schizophrenic Patients. BMC Psychiatry.

[70] Pavăl D., Gherghel-Pavăl N., Căpățînă O.O., Stan A., Micluția I.V., Giné-Servén E. (2023). The Importance of Cerebrospinal Fluid Investigation in First-Episode Psychosis. Yale J. Biol. Med..

[71] Bernstein H.-G., Dobrowolny H., Keilhoff G., Bogerts B., Steiner J. (2018). Reduced Density of DISC1 Expressing Astrocytes in the Dentate Gyrus but Not in the Subventricular Zone in Schizophrenia. Neuropsychopharmacology.

[72] Laricchiuta D., Papi M., Decandia D., Panuccio A., Cutuli D., Peciccia M., Mazzeschi C., Petrosini L. (2024). The Role of Glial Cells in Mental Illness: A Systematic Review on Astroglia and Microglia as Potential Players in Schizophrenia and Its Cognitive and Emotional Aspects. Front. Cell Neurosci..

[73] Daverey A., Agrawal S.K. (2016). Curcumin Alleviates Oxidative Stress and Mitochondrial Dysfunction in Astrocytes. Neuroscience.

[74] Najjar S., Pahlajani S., De Sanctis V., Stern J.N.H., Najjar A., Chong D. (2017). Neurovascular Unit Dysfunction and Blood–Brain Barrier Hyperpermeability Contribute to Schizophrenia Neurobiology: A Theoretical Integration of Clinical and Experimental Evidence. Front. Psychiatry.

[75] Pollak T.A., Lennox B.R., Müller S., Benros M.E., Prüss H., Tebartz van Elst L., Klein H., Steiner J., Frodl T., Bogerts B. (2020). Autoimmune Psychosis: An International Consensus on an Approach to the Diagnosis and Management of Psychosis of Suspected Autoimmune Origin. Lancet Psychiatry.

[76] Stanca S., Rossetti M., Bokulic Panichi L., Bongioanni P. (2024). The Cellular Dysfunction of the Brain–Blood Barrier from Endothelial Cells to Astrocytes: The Pathway towards Neurotransmitter Impairment in Schizophrenia. Int. J. Mol. Sci..

[77] Steiner J., Schiltz K., Walter M., Wunderlich M.T., Keilhoff G., Brisch R., Bielau H., Bernstein H.-G., Bogerts B., Schroeter M.L. (2010). S100B Serum Levels Are Closely Correlated with Body Mass Index: An Important Caveat in Neuropsychiatric Research. Psychoneuroendocrinology.

[78] Aleksovska K., Leoncini E., Bonassi S., Cesario A., Boccia S., Frustaci A. (2014). Systematic Review and Meta-Analysis of Circulating S100B Blood Levels in Schizophrenia. PLoS ONE.

[79] Schümberg K., Polyakova M., Steiner J., Schroeter M.L. (2016). Serum S100B Is Related to Illness Duration and Clinical Symptoms in Schizophrenia—A Meta-Regression Analysis. Front. Cell Neurosci..

[80] Michinaga S., Koyama Y. (2019). Dual Roles of Astrocyte-Derived Factors in Regulation of Blood-Brain Barrier Function after Brain Damage. Int. J. Mol. Sci..

[81] Giotakos O. (2019). Is Psychosis a Dysmyelination-Related Information-Processing Disorder?. Psychiatriki.

[82] Vanes L.D., Mouchlianitis E., Barry E., Patel K., Wong K., Shergill S.S. (2019). Cognitive Correlates of Abnormal Myelination in Psychosis. Sci. Rep..

[83] Zhang R., He J., Zhu S., Zhang H., Wang H., Adilijiang A., Kong L., Wang J., Kong J., Tan Q. (2012). Myelination Deficit in a Phencyclidine-Induced Neurodevelopmental Model of Schizophrenia. Brain Res..

[84] Mighdoll M.I., Tao R., Kleinman J.E., Hyde T.M. (2015). Myelin, Myelin-Related Disorders, and Psychosis. Schizophr. Res..

[85] Madsen P.M., Desu H.L., de Rivero Vaccari J.P., Florimon Y., Ellman D.G., Keane R.W., Clausen B.H., Lambertsen K.L., Brambilla R. (2020). Oligodendrocytes Modulate the Immune-Inflammatory Response in EAE via TNFR2 Signaling. Brain Behav. Immun..

[86] Boccazzi M., Raffaele S., Fumagalli M. (2022). Not Only Myelination: The Immune-Inflammatory Functions of Oligodendrocytes. Neural. Regen. Res..

[87] Domingues H.S., Portugal C.C., Socodato R., Relvas J.B. (2016). Oligodendrocyte, Astrocyte, and Microglia Crosstalk in Myelin Development, Damage, and Repair. Front. Cell Dev. Biol..

[88] Sun M., You H., Hu X., Luo Y., Zhang Z., Song Y., An J., Lu H. (2023). Microglia-Astrocyte Interaction in Neural Development and Neural Pathogenesis. Cells.

[89] Sheng J.A., Bales N.J., Myers S.A., Bautista A.I., Roueinfar M., Hale T.M., Handa R.J. (2020). The Hypothalamic-Pituitary-Adrenal Axis: Development, Programming Actions of Hormones, and Maternal-Fetal Interactions. Front. Behav. Neurosci..

[90] Mayo D., Corey S., Kelly L.H., Yohannes S., Youngquist A.L., Stuart B.K., Niendam T.A., Loewy R.L. (2017). The Role of Trauma and Stressful Life Events among Individuals at Clinical High Risk for Psychosis: A Review. Front. Psychiatry.

[91] Kazi S.E., Hoque S. (2021). Acute Psychosis Following Corticosteroid Administration. Cureus.

[92] Mikulska J., Juszczyk G., Gawronska-Grzywacz M., Herbet M. (2021). HPA Axis in the Pathomechanism of Depression and Schizophrenia: New Therapeutic Strategies Based on Its Participation. Brain Sci..

[93] Rutigliano G., Chaumette B., Seeman M.V. (2020). Editorial: Psychoneuroendocrinology of Psychosis Disorders. Front. Psychiatry..

[94] Sudo N., Chida Y., Aiba Y., Sonoda J., Oyama N., Yu X.-N., Kubo C., Koga Y. (2004). Postnatal Microbial Colonization Programs the Hypothalamic-Pituitary-Adrenal System for Stress Response in Mice. J. Physiol..

[95] Sharon G., Sampson T.R., Geschwind D.H., Mazmanian S.K. (2016). The Central Nervous System and the Gut Microbiome. Cell.

[96] Kelly J.R., Minuto C., Cryan J.F., Clarke G., Dinan T.G. (2021). The Role of the Gut Microbiome in the Development of Schizophrenia. Schizophr. Res..

[97] Murray N., Ghomi R.H., Nemani K., O’Connor K., Hyland N., Stanton C. (2024). The Influence of Gut Microbiota in Psychosis. The Gut-Brain Axis.

[98] Foster J.A., McVey Neufeld K.A. (2013). Gut-Brain Axis: How the Microbiome Influences Anxiety and Depression. Trends Neurosci..

[99] MacKay M., Yang B.H., Dursun S.M., Baker G.B. (2024). The Gut-Brain Axis and the Microbiome in Anxiety Disorders, Post-Traumatic Stress Disorder and Obsessive-Compulsive Disorder. Curr. Neuropharmacol..

[100] Kraeuter A.K., Phillips R., Sarnyai Z. (2020). The Gut Microbiome in Psychosis From Mice to Men: A Systematic Review of Preclinical and Clinical Studies. Front. Psychiatry.

[101] Zhu F., Guo R., Wang W., Ju Y., Wang Q., Ma Q., Sun Q., Fan Y., Xie Y., Yang Z. (2020). Transplantation of Microbiota from Drug-Free Patients with Schizophrenia Causes Schizophrenia-like Abnormal Behaviors and Dysregulated Kynurenine Metabolism in Mice. Mol. Psychiatry.

[102] Zheng P., Zeng B., Liu M., Chen J., Pan J., Han Y., Liu Y., Cheng K., Zhou C., Wang H. (2019). The Gut Microbiome from Patients with Schizophrenia Modulates the Glutamate-Glutamine-GABA Cycle and Schizophrenia-Relevant Behaviors in Mice. Sci. Adv..

[103] McGuinness A.J., Davis J.A., Dawson S.L., Loughman A., Collier F., O’Hely M., Simpson C.A., Green J., Marx W., Hair C. (2022). A Systematic Review of Gut Microbiota Composition in Observational Studies of Major Depressive Disorder, Bipolar Disorder and Schizophrenia. Mol. Psychiatry.

[104] Nocera A., Nasrallah H.A. (2022). The Association of the Gut Microbiota with Clinical Features in Schizophrenia. Behav. Sci..

[105] Nuncio-Mora L., Lanzagorta N., Nicolini H., Sarmiento E., Ortiz G., Sosa F., Genis-Mendoza A.D. (2023). The Role of the Microbiome in First Episode of Psychosis. Biomedicines.

[106] Erny D., Hrabě de Angelis A.L., Jaitin D., Wieghofer P., Staszewski O., David E., Keren-Shaul H., Mahlakoiv T., Jakobshagen K., Buch T. (2015). Host Microbiota Constantly Control Maturation and Function of Microglia in the CNS. Nat. Neurosci..

[107] Misiak B., Łoniewski I., Marlicz W., Frydecka D., Szulc A., Rudzki L., Samochowiec J. (2020). The HPA Axis Dysregulation in Severe Mental Illness: Can We Shift the Blame to Gut Microbiota?. Prog. Neuropsychopharmacol. Biol. Psychiatry.

[108] Yuan X., Kang Y., Zhuo C., Huang X.F., Song X. (2019). The Gut Microbiota Promotes the Pathogenesis of Schizophrenia via Multiple Pathways. Biochem. Biophys. Res. Commun..

[109] Zhao Y.-F., Wei D.-N., Tang Y. (2021). Gut Microbiota Regulate Astrocytic Functions in the Brain: Possible Therapeutic Consequences. Curr. Neuropharmacol..

[110] Hoban A.E., Stilling R.M., Ryan F.J., Shanahan F., Dinan T.G., Claesson M.J., Clarke G., Cryan J.F. (2016). Regulation of Prefrontal Cortex Myelination by the Microbiota. Transl. Psychiatry.

[111] O’Mahony S.M., Marchesi J.R., Scully P., Codling C., Ceolho A.-M., Quigley E.M.M., Cryan J.F., Dinan T.G. (2009). Early Life Stress Alters Behavior, Immunity, and Microbiota in Rats: Implications for Irritable Bowel Syndrome and Psychiatric Illnesses. Biol. Psychiatry.

[112] Bailey M.T., Coe C.L. (1999). Maternal Separation Disrupts the Integrity of the Intestinal Microflora in Infant Rhesus Monkeys. Dev. Psychobiol..

[113] Ait-Belgnaoui A., Payard I., Rolland C., Harkat C., Braniste V., Théodorou V., Tompkins T.A. (2018). Bifidobacterium Longum and Lactobacillus Helveticus Synergistically Suppress Stress-Related Visceral Hypersensitivity Through Hypothalamic-Pituitary-Adrenal Axis Modulation. J. Neurogastroenterol. Motil..

[114] Nagy-Grócz G., Spekker E., Vécsei L. (2024). Kynurenines, Neuronal Excitotoxicity, and Mitochondrial Oxidative Stress: Role of the Intestinal Flora. Int. J. Mol. Sci..

[115] Gilmore J.H., Knickmeyer R.C., Gao W. (2018). Imaging Structural and Functional Brain Development in Early Childhood. Nat. Rev. Neurosci..

[116] Chiappelli J., Postolache T.T., Kochunov P., Rowland L.M., Wijtenburg S.A., Shukla D.K., Tagamets M., Du X., Savransky A., Lowry C.A. (2016). Tryptophan Metabolism and White Matter Integrity in Schizophrenia. Neuropsychopharmacology.

[117] Tsamakis K., Galinaki S., Alevyzakis E., Hortis I., Tsiptsios D., Kollintza E., Kympouropoulos S., Triantafyllou K., Smyrnis N., Rizos E. (2022). Gut Microbiome: A Brief Review on Its Role in Schizophrenia and First Episode of Psychosis. Microorganisms.

[118] Caldara M., Marmiroli N. (2021). Antimicrobial Properties of Antidepressants and Antipsychotics—Possibilities and Implications. Pharmaceuticals.

[119] Seeman M.V. (2023). What Is the Significance of the Impact of Antipsychotics on the Gut Microbiome?. Expert. Opin. Drug Metab. Toxicol..

[120] Bretler T., Weisberg H., Koren O., Neuman H. (2019). The Effects of Antipsychotic Medications on Microbiome and Weight Gain in Children and Adolescents. BMC Med..

[121] Vasileva S.S., Tucker J., Siskind D., Eyles D. (2022). Does the Gut Microbiome Mediate Antipsychotic-Induced Metabolic Side Effects in Schizophrenia?. Expert. Opin. Drug Saf..

[122] Seeman M.V. (2021). The Gut Microbiome and Antipsychotic Treatment Response. Behav. Brain Res..

[123] Murray A.J., Rogers J.C., Katshu M.Z.U.H., Liddle P.F., Upthegrove R. (2021). Oxidative Stress and the Pathophysiology and Symptom Profile of Schizophrenia Spectrum Disorders. Front. Psychiatry.

[124] Lanté F., Meunier J., Guiramand J., Maurice T., Cavalier M., de Jesus Ferreira M.-C., Aimar R., Cohen-Solal C., Vignes M., Barbanel G. (2007). Neurodevelopmental Damage after Prenatal Infection: Role of Oxidative Stress in the Fetal Brain. Free Radic. Biol. Med..

[125] Fraguas D., Díaz-Caneja C.M., Ayora M., Hernández-Álvarez F., Rodríguez-Quiroga A., Recio S., Leza J.C., Arango C. (2019). Oxidative Stress and Inflammation in First-Episode Psychosis: A Systematic Review and Meta-Analysis. Schizophr. Bull..

[126] Jîtcă G., Ősz B.E., Tero-Vescan A., Vari C.E. (2021). Psychoactive Drugs—From Chemical Structure to Oxidative Stress Related to Dopaminergic Neurotransmission. A Review. Antioxidants.

[127] Miyazaki I., Asanuma M. (2008). Dopaminergic Neuron-Specific Oxidative Stress Caused by Dopamine Itself. Acta Med. Okayama.

[128] Rambaud V., Marzo A., Chaumette B. (2022). Oxidative Stress and Emergence of Psychosis. Antioxidants.

[129] Grima G. (2003). Dopamine-Induced Oxidative Stress in Neurons with Glutathione Deficit: Implication for Schizophrenia. Schizophr. Res..

[130] Cuenod M., Steullet P., Cabungcal J.H., Dwir D., Khadimallah I., Klauser P., Conus P., Do K.Q. (2022). Caught in Vicious Circles: A Perspective on Dynamic Feed-Forward Loops Driving Oxidative Stress in Schizophrenia. Mol. Psychiatry.

[131] Vallée A. (2022). Neuroinflammation in Schizophrenia: The Key Role of the WNT/β-Catenin Pathway. Int. J. Mol. Sci..

[132] Hardingham G.E., Do K.Q. (2016). Linking Early-Life NMDAR Hypofunction and Oxidative Stress in Schizophrenia Pathogenesis. Nat. Rev. Neurosci..

[133] Steullet P., Cabungcal J.H., Monin A., Dwir D., O’Donnell P., Cuenod M., Do K.Q. (2016). Redox Dysregulation, Neuroinflammation, and NMDA Receptor Hypofunction: A “Central Hub” in Schizophrenia Pathophysiology?. Schizophr. Res..

[134] Rapaport M.H., Delrahim K.K., Bresee C.J., Maddux R.E., Ahmadpour O., Dolnak D. (2005). Celecoxib Augmentation of Continuously Ill Patients with Schizophrenia. Biol. Psychiatry.

[135] Magalhães P.V.S., Dean O., Andreazza A.C., Berk M., Kapczinski F. (2016). Antioxidant Treatments for Schizophrenia. Cochrane Database Syst. Rev..

[136] Wong-Riley M.T.T. (1989). Cytochrome Oxidase: An Endogenous Metabolic Marker for Neuronal Activity. Trends Neurosci..

[137] Hüttemann M., Kadenbach B., Grossman L.I. (2001). Mammalian Subunit IV Isoforms of Cytochrome c Oxidase. Gene.

[138] Roberts R.C. (2021). Mitochondrial Dysfunction in Schizophrenia: With a Focus on Postmortem Studies. Mitochondrion.

[139] Hjelm B.E., Rollins B., Mamdani F., Lauterborn J.C., Kirov G., Lynch G., Gall C.M., Sequeira A., Vawter M.P. (2015). Evidence of Mitochondrial Dysfunction within the Complex Genetic Etiology of Schizophrenia. Complex Psychiatry.

[140] Rajasekaran A., Venkatasubramanian G., Berk M., Debnath M. (2015). Mitochondrial Dysfunction in Schizophrenia: Pathways, Mechanisms and Implications. Neurosci. Biobehav. Rev..

[141] Prabakaran S., Swatton J.E., Ryan M.M., Huffaker S.J., Huang J.-J., Griffin J.L., Wayland M., Freeman T., Dudbridge F., Lilley K.S. (2004). Mitochondrial Dysfunction in Schizophrenia: Evidence for Compromised Brain Metabolism and Oxidative Stress. Mol. Psychiatry.

[142] Looney J.M., Childs H.M. (1934). The Lactic Acid and Glutathione Content of the Blood of Schizophrenic Patients 1. J. Clin. Investig..

[143] Stork C., Renshaw P.F. (2005). Mitochondrial Dysfunction in Bipolar Disorder: Evidence from Magnetic Resonance Spectroscopy Research. Mol. Psychiatry.

[144] Regenold W.T., Phatak P., Marano C.M., Sassan A., Conley R.R., Kling M.A. (2009). Elevated Cerebrospinal Fluid Lactate Concentrations in Patients with Bipolar Disorder and Schizophrenia: Implications for the Mitochondrial Dysfunction Hypothesis. Biol. Psychiatry.

[145] Hastings T.G., Zigmond M.J. (1994). Identification of Catechol-Protein Conjugates in Neostriatal Slices Incubated with [^3^H]Dopamine: Impact of Ascorbic Acid and Glutathione. J. Neurochem..

[146] Masserano J.M., Gong L., Kulaga H., Baker I., Wyatt R.J. (1996). Dopamine Induces Apoptotic Cell Death of a Catecholaminergic Cell Line Derived from the Central Nervous System. Mol. Pharmacol..

[147] Ben-Shachar D., Zuk R., Gazawi H., Ljubuncic P. (2004). Dopamine Toxicity Involves Mitochondrial Complex I Inhibition: Implications to Dopamine-Related Neuropsychiatric Disorders. Biochem. Pharmacol..

[148] Berk M., Plein H., Belsham B. (2000). The Specificity of Platelet Glutamate Receptor Supersensitivity in Psychotic Disorders. Life Sci..

[149] Bitanihirwe B.K.Y., Woo T.-U.W. (2011). Oxidative Stress in Schizophrenia: An Integrated Approach. Neurosci. Biobehav. Rev..

[150] O’Donnell P., Do K.Q., Arango C. (2014). Oxidative/Nitrosative Stress in Psychiatric Disorders: Are We There Yet?. Schizophr. Bull..

[151] Halliwell B. (2006). Reactive Species and Antioxidants. Redox Biology Is a Fundamental Theme of Aerobic Life. Plant Physiol..

[152] Wagner K.R., Kleinholz M., Myers R.E. (1990). Delayed Decreases in Specific Brain Mitochondrial Electron Transfer Complex Activities and Cytochrome Concentrations Following Anoxia/Ischemia. J. Neurol. Sci..

[153] Singh O., Chakraborty I., Dasgupta A., Datta S. (2008). A Comparative Study of Oxidative Stress and Interrelationship of Important Antioxidants in Haloperidol and Olanzapine Treated Patients Suffering from Schizophrenia. Indian. J. Psychiatry.

[154] Raffa M., Mechri A., Othman L.B., Fendri C., Gaha L., Kerkeni A. (2009). Decreased Glutathione Levels and Antioxidant Enzyme Activities in Untreated and Treated Schizophrenic Patients. Prog. Neuropsychopharmacol. Biol. Psychiatry.

[155] Chowdari K.V., Bamne M.N., Nimgaonkar V.L. (2011). Genetic Association Studies of Antioxidant Pathway Genes and Schizophrenia. Antioxid. Redox Signal.

[156] Gubert C., Stertz L., Pfaffenseller B., Panizzutti B.S., Rezin G.T., Massuda R., Streck E.L., Gama C.S., Kapczinski F., Kunz M. (2013). Mitochondrial Activity and Oxidative Stress Markers in Peripheral Blood Mononuclear Cells of Patients with Bipolar Disorder, Schizophrenia, and Healthy Subjects. J. Psychiatr. Res..

[157] Naik E., Dixit V.M. (2011). Mitochondrial Reactive Oxygen Species Drive Proinflammatory Cytokine Production. J. Exp. Med..

[158] Al-Asmari A., Khan M.W. (2014). Inflammation and Schizophrenia: Alterations in Cytokine Levels and Perturbation in Antioxidative Defense Systems. Hum. Exp. Toxicol..

[159] Harrison P.J. (2015). Recent Genetic Findings in Schizophrenia and Their Therapeutic Relevance. J. Psychopharmacol..

[160] Snyder M.A., Gao W.-J. (2020). NMDA Receptor Hypofunction for Schizophrenia Revisited: Perspectives from Epigenetic. Mechanisms. Schizophr. Res..

[161] Harrison P.J., Weinberger D.R. (2005). Schizophrenia Genes, Gene Expression, and Neuropathology: On the Matter of Their Convergence. Mol. Psychiatry.

[162] Alam R., Abdolmaleky H.M., Zhou J. (2017). Microbiome, Inflammation, Epigenetic Alterations, and Mental Diseases. Am. J. Med. Genet. Part B Neuropsychiatr. Genetics.

[163] Guidotti A., Auta J., Davis J.M., Gerevini V.D., Dwivedi Y., Grayson D.R., Impagnatiello F., Pandey G., Pesold C., Sharma R. (2000). Decrease in Reelin and Glutamic Acid Decarboxylase67 (GAD67) Expression in Schizophrenia and Bipolar Disorder. Arch. Gen. Psychiatry.

[164] Negrón-Oyarzo I., Lara-Vásquez A., Palacios-García I., Fuentealba P., Aboitiz F. (2016). Schizophrenia and Reelin: A Model Based on Prenatal Stress to Study Epigenetics, Brain Development and Behavior. Biol. Res..

[165] Li M., Xiao L., Chen X. (2022). Histone Acetylation and Methylation Underlie Oligodendroglial and Myelin Susceptibility in Schizophrenia. Front. Cell Neurosci..

[166] Smigielski L., Jagannath V., Rössler W., Walitza S., Grünblatt E. (2020). Epigenetic Mechanisms in Schizophrenia and Other Psychotic Disorders: A Systematic Review of Empirical Human Findings. Mol. Psychiatry.

[167] Wawrzczak-Bargieła A., Bilecki W., Maćkowiak M. (2023). Epigenetic Targets in Schizophrenia Development and Therapy. Brain Sci..

[168] Goff D.C., Romero K., Paul J., Mercedes Perez-Rodriguez M., Crandall D., Potkin S.G. (2016). Biomarkers for Drug Development in Early Psychosis: Current Issues and Promising Directions. Eur. Neuropsychopharmacol..

[169] De Picker L., Fransen E., Coppens V., Timmers M., de Boer P., Oberacher H., Fuchs D., Verkerk R., Sabbe B., Morrens M. (2020). Immune and Neuroendocrine Trait and State Markers in Psychotic Illness: Decreased Kynurenines Marking Psychotic Exacerbations. Front. Immunol..

[170] Yadav M., Kumar N., Kumar A., Jindal D.K., Dahiya M. (2021). Possible Biomarkers and Contributing Factors of Psychosis: A Review. Curr. Pharmacol. Rep..

[171] Seitz-Holland J., Alemán-Gómez Y., Cho K.I.K., Pasternak O., Cleusix M., Jenni R., Baumann P.S., Klauser P., Conus P., Hagmann P. (2024). Matrix Metalloproteinase 9 (MMP-9) Activity, Hippocampal Extracellular Free Water, and Cognitive Deficits Are Associated with Each Other in Early Phase Psychosis. Neuropsychopharmacology.

[172] Yue W., Huang H., Duan J. (2022). Potential Diagnostic Biomarkers for Schizophrenia. Med. Rev..

[173] Yu H., Ni P., Zhao L., Tian Y., Li M., Li X., Wei W., Wei J., Deng W., Du X. (2023). Decreased Plasma Neuropeptides in First-Episode Schizophrenia, Bipolar Disorder, Major Depressive Disorder: Associations with Clinical Symptoms and Cognitive Function. Front. Psychiatry.

[174] Minichino A., Brondino N., Solmi M., Del Giovane C., Fusar-Poli P., Burnet P., Cipriani A., Lennox B.R. (2021). The Gut-Microbiome as a Target for the Treatment of Schizophrenia: A Systematic Review and Meta-Analysis of Randomised Controlled Trials of Add-on Strategies. Schizophr. Res..

[175] Woodberry K.A., Shapiro D.I., Bryant C., Seidman L.J. (2016). Progress and Future Directions in Research on the Psychosis Prodrome. Harv. Rev. Psychiatry.

[176] Potkin S.G., Kane J.M., Correll C.U., Lindenmayer J.-P., Agid O., Marder S.R., Olfson M., Howes O.D. (2020). The Neurobiology of Treatment-Resistant Schizophrenia: Paths to Antipsychotic Resistance and a Roadmap for Future Research. NPJ Schizophr..

[177] Chen E.Y.H., Wong S.M.Y. (2024). Unique Challenges in Biomarkers for Psychotic Disorders. Brain Sci..

[178] Lynch C.M.K., O’Riordan K.J., Clarke G., Cryan J.F., Pimental M., Mathur R., Barlow G.M. (2023). Gut Microbes: The Gut Brain Connection. Clinical Understanding of the Human Gut Microbiome.

